# Nonlinear dynamics of cardiovascular ageing

**DOI:** 10.1016/j.physrep.2009.12.003

**Published:** 2010-03

**Authors:** Y. Shiogai, A. Stefanovska, P.V.E. McClintock

**Affiliations:** a Physics Department, Lancaster University, Lancaster LA1 4YB, UK; b Faculty of Electrical Engineering, University of Ljubljana, Ljubljana, Slovenia

**Keywords:** Coupled oscillators, Wavelet transform, Synchronization, Ageing, Complexity, Phase dynamics, Heart rate variability, Iontophoresis, Endothelial function, Blood flow

## Abstract

The application of methods drawn from nonlinear and stochastic dynamics to the analysis of cardiovascular time series is reviewed, with particular reference to the identification of changes associated with ageing. The natural variability of the heart rate (HRV) is considered in detail, including the respiratory sinus arrhythmia (RSA) corresponding to modulation of the instantaneous cardiac frequency by the rhythm of respiration. HRV has been intensively studied using traditional spectral analyses, e.g. by Fourier transform or autoregressive methods, and, because of its complexity, has been used as a paradigm for testing several proposed new methods of complexity analysis. These methods are reviewed. The application of time–frequency methods to HRV is considered, including in particular the wavelet transform which can resolve the time-dependent spectral content of HRV. Attention is focused on the cardio-respiratory interaction by introduction of the respiratory frequency variability signal (RFV), which can be acquired simultaneously with HRV by use of a respiratory effort transducer. Current methods for the analysis of interacting oscillators are reviewed and applied to cardio-respiratory data, including those for the quantification of synchronization and direction of coupling. These reveal the effect of ageing on the cardio-respiratory interaction through changes in the mutual modulation of the instantaneous cardiac and respiratory frequencies. Analyses of blood flow signals recorded with laser Doppler flowmetry are reviewed and related to the current understanding of how endothelial-dependent oscillations evolve with age: the inner lining of the vessels (the endothelium) is shown to be of crucial importance to the emerging picture. It is concluded that analyses of the complex and nonlinear dynamics of the cardiovascular system can illuminate the mechanisms of blood circulation, and that the heart, the lungs and the vascular system function as a single entity in dynamical terms. Clear evidence is found for dynamical ageing.

## 1 Introduction

In this paper, we review the effects of ageing on the cardiovascular system, based on ideas drawn from nonlinear dynamics. We aim to cover the major achievements in this field, including results recently obtained in Ljubljana and Lancaster.

We start by outlining the motivation, structure and content of the review. It has long been known that cardiovascular signals contain a number of oscillatory components that are not exactly periodic. To put it differently, their periods (frequencies) fluctuate with time. For example, heart rate variability (HRV) has in itself provided a major topic of discussion. We introduce one of the statistical approaches to HRV in Section 3. However, in order to understand the variability of the cardiovascular system, discussion of a single source is insufficient because the cardiovascular system is composed of many different physiological components (subsystems) and it is the effects of their mutual interaction that combine to produce HRV. This is demonstrated in Section 4, revealed by results obtained using the wavelet transform. In Section 5, we discuss the cardio-respiratory interaction in terms of phase synchronization. To set the scene for these later discussions, we summarize the basic principles of phase dynamics in Section 2. For readers who are unfamiliar with the physiological aspects of the research, we provide Appendices A on the cardiovascular system and B on how measurements of cardiovascular signals are conducted. Appendix C provides details of the statistical methods used in the group data analyses.

Before embarking on the central topics, however, we first summarize their historical background in order to set the review in context.

### 1.1 Cardiovascular signals in context

Cardiovascular signals carry information that reflects ongoing processes that normally occur unseen, within the interior of the body. They can be used to characterize the *state* of the system, including the diagnosis of incipient pathophysiological conditions before symptoms become obvious. A well-known example is the electrocardiogram (ECG) signal, representing the electrical activity of the heart. ECG measurements have been used for diagnostic purposes for almost a century. For the first several decades of such measurements, attention was focused mainly on the detailed *shape* of the approximately periodic pulses seen in the signal. The physiological relationships that could be drawn from the data were restricted to static values because only chart recorders were available.

With the advent of computers, starting in the 1960s, it became possible to sample physiological variables in real time and to store data for analysis. The resultant time series (signals) immediately introduced a need for tools for studying the dynamical properties of the underlying physiological processes. Because of the complexity of the time series, the tools developed for spectral analysis were applied mainly with the aim of filtering out the noise, thereby reducing the complexity. Various methods of linear filtering were introduced, as was also a fast algorithm for calculation of the Fourier transform (now well known as the fast Fourier transform, or FFT). Application of the FFT to the most studied cardiovascular signal, the ECG, quickly showed that it possesses oscillatory components [1]. In their pioneering work Hyndman, Kitney and Sayers [2] pointed to the generally oscillatory nature of physiological control systems. These two studies initiated a large area of research into the oscillatory nature of cardiovascular functions based on frequency and time–frequency methods including FFT [3] parametric spectral estimation [4] and wavelet spectra [5–7].

The investigation of deterministic chaotic dynamics and, in particular, the introduction of measures to quantify fractal dynamics triggered an avalanche of new studies of cardiovascular dynamics. The pioneering algorithm of Grassberger and Proccacia [8] motivated a large number of applications, and chaotic behaviour was proposed as a possible scenario [9,10]. Several methods based on statistical physics were proposed. Scaling properties [11–15], multifractal properties [16,17], and the 1/f spectra [18–20] of heart rate variability (HRV), were all discussed.

The approach based on coupled nonlinear oscillators was to some extent developed separately. It was marked by two major milestones: introduction of the concept of entrainment within an ensemble of oscillators by Winfree [21]; and its analysis by Kuramoto [22] using a phase model. After Winfree had gone further into the theory of the geometry of biological time [23], Kuramoto generalized the phase dynamics approach [24] by reducing the degrees of freedom of the original dynamical system. For this to work, the original dynamics should be perturbed weakly by noise, by an external force, or by coupling to dynamics with a limit cycle orbit. The latter describes dissipative systems and the form of the phase dynamics is not dependent on the form of the original model. Numerous researchers contributed to the development of the theory, and the model was further generalized by Strogatz [25]. Because of its universality and simplicity, phase dynamics can be applied quite generally to oscillatory phenomena in dissipative systems. It was this body of work that subsequently motivated the introduction of the theory of phase synchronization, facilitating studies of the interactions between coupled nonlinear and chaotic oscillators [26]. Coupled oscillators were proposed as a possible description of the dynamics of the cardiovascular system [5] and synchronization between cardiac and respiratory oscillations, and their mutual modulation, were examined with particular care [7,27–33]. The emerging picture motivated additional studies, and several methods for analysis of the direction of coupling among interacting oscillatory processes have recently been proposed [34–37].

The mystery of ageing has continued to intrigue, giving rise to studies in all areas of physiology. The relationship between HRV and ageing was soon appreciated [38–40]. Goldberger and co-workers were the first to study the dynamics of cardiovascular ageing, using measures drawn from statistical physics to show that the complexity of cardiovascular dynamics decreases with ageing [41–44]. Furthermore, randomness in the heartbeat time series [45], and loss of time irreversibility [46], were shown to occur with ageing. Ageing has also been characterized by a decrease in endothelial-related vasodilation [47,48] and, very recently, by an insufficiency of the sympathetic nervous system to cope dynamically with various environmental stimuli [49].

### 1.2 Coupled nonlinear oscillators and the cardiovascular system

Coupled oscillators have been investigated by many physicists, in part because the emergence of synchronization has similarities to phase transition phenomena, which had been studied earlier in many contexts. The synchronization transition was analysed by the application of mean field theory to globally coupled ensembles of oscillators, in which each oscillator is coupled to all the other oscillators equally under a sine coupling function (the Kuramoto model). The stability of the macroscopic oscillation (synchronized solution) was addressed by Crawford and Strogatz [50–52], and the coupled function was extended by Sakaguchi [53]. Not only global coupling, but also local coupling in which a given oscillator couples only to its nearest neighbours, and which is equivalent to the diffusion coupling in a continuous system, have been studied extensively, e.g. in the form of the Ginzburg–Landau equation [24]. Kuramoto also suggested another form of coupling, intermediate between local and global, known as nonlocal coupling [54,55]. It has a finite coupling distance so an oscillator can interact not only with its nearest neighbours, but also with other nearby oscillators. It is more realistic than global coupling because a given oscillator cannot in reality interact with all the others because of the finite coupling distance. Compared to local and global coupling, which have been studied widely, nonlocal coupling has not been studied very much to date. But this model is expected to be useful because its coupling length is adjustable to match reality. It is expected to encompass interesting phenomena that are as yet undiscovered. Studies of nonlocal coupling include [56–60].

In the human cardiovascular system, there are many phenomena to which the concept of entrainment, or synchronization, of coupled oscillators can be applied. One of them is the emergence of macroscopic oscillations through the entrainment of the individual microscopic oscillations of individual cells which, in the uncoupled state, would have slightly different frequencies. For example, it is well known that the heart has pacemaker cells to which other cells are entrained. It is also reported that the initiation of vasomotion requires the synchronization of Ca^2+^ release from the sarcoplasmic reticulum [61]. Entrainment can also arise through the interaction of macroscopic oscillators of different physiological origin. In the latter case, coupled oscillators were proposed as a possible description of the dynamics of the cardiovascular system [5]: synchronization between cardiac and respiratory oscillations, and their mutual modulation, have been examined with particular care [7,27–33]. The emerging picture motivated additional studies, and several methods have recently been proposed for analysis of the direction of coupling among interacting oscillatory processes [34–37]. Interactions between the cardiovascular oscillations and brain waves have been also studied by using the concepts of coupled oscillators and directionality [62,63]. The notion of phase dynamics can be useful in terms not only of phase synchronization but also of phase resetting [21]. For example, the annihilation of pacemaker activity in cardiac tissues was observed [64] via phase resetting. The authors used a current pulse to stimulate SA nodal pacemaker cells (see A.3.2), and observed phase resetting phenomena. If the timing and amplitude were appropriate, the autonomous oscillatory activity stopped. Spiral waves during cardiac fibrillation can be terminated by shock-induced phase resetting [65]: such spiral waves, rotating around singularities in the heart, known as ventricular fibrillation, can lead to death because the heart cannot then pump the blood properly. The latter represents a successful application of phase dynamics to clinical medicine.

### 1.3 Time-invariant complexity analysis of heart rate variability (HRV)

The investigation of deterministic chaotic dynamics, and in particular the introduction of measures to quantify the complexity of fractal dynamics, triggered an avalanche of new work, including cardiovascular studies. Hurst introduced what is now known as the Hurst exponent to quantify a scaling property when he investigated problems related to water storage in the Nile [66,67]. Mandelbrot and Wallis examined and elaborated the method further [68–74]. Feder gives an excellent overview of the history, theory and applications, and adds some more statistical experiments in [75]. Although estimation of the Hurst exponent was originally developed in hydrology, modern techniques for estimating the Hurst exponent come from fractal mathematics. The mathematics and images derived from fractal geometry exploded during the 1970s and 1980s. A fractal object is composed of sub-units and sub-sub-units on multiple levels that resemble the structure of the whole object (self-similarity) and it has a fractional dimension. Chaotic dynamics is often associated with a strange attractor that can be characterized by its fractal dimensionality D [76]. This dimension of a chaotic system is one of the ways to measure complexity. The pioneering algorithm introduced by Grassberger and Proccacia enabled the ‘strangeness’ of an attractor to be calculated in an easier way [8] and motivated a large number of applications. Another method for the measurement of complexity based on an entropy, was also proposed by Grassberger and Proccacia [77]. Ways to compute the approximate dimension and approximate entropy were suggested by Kaplan et al. [41]. Chaotic behaviour was proposed as a possible scenario [9,10]. Several methods based on statistical physics were introduced. Scaling properties [11–15], multifractal properties [16,17], and the 1/f spectra [18–20] of heart rate variability (HRV) were also discussed in considerable detail.

On the other hand, the heart rate is known to have characteristics that differ between healthy subjects and subjects with heart disease [44]. The heart rate of healthy subjects is far from being a homeostatic constant state and has visually apparent non-stationarity, whereas the heart rate in heart disease is associated with the emergence of excessive regularity or uncorrelated randomness. A constant heart rate was observed in the case of a coma [78], demonstrating again that some measure of irregularity is needed for health. These features are thought to be related to fractal and nonlinear properties. Quantifying the complexity of healthy heart rate, and detecting its alterations with disease and with ageing represent major challenges in physiology.

New methods have been developed to replace the traditional approaches used for stationary signals, such as power spectral and autocorrelation analysis. They can quantify accurately the ‘long range’ correlation (see definitions in Section 3.3) in non-stationary signals: these include detrended fluctuation analysis (DFA) [79,80] and the detrended moving average method (DMA) [81–83]. They too are based on the idea of a fractal in nonlinear theory. The fractal concept is extended to time series so that we can see the self-similar properties on different time scales. DFA is a method used to quantify the fractal correlation in time series by filtering out polynomial trends as discussed below in Section 3. To avoid the assumption that the trend is necessarily polynomial, the DMA method was introduced. It estimates the correlation properties of non-stationary signals, the probability distribution, and other characteristic of stochastic processes, without any assumption of trends. These methods have been applied to financial [82], physiological [84–86] and biological signals [87].

It has been suggested that the HRV of healthy subjects shows self-similar (scale-invariant) fluctuations over a wide range of time scales, and that disease and ageing make HRV less complex (with higher regularity and predictability). On the basis of DFA analysis, it was reported that complexity decreases with increasing age [49,88]. The physiological background to the loss of complexity with age has been studied extensively. It has not been fully elucidated, but changes in the balance between two branches (sympathetic and vagal) of the autonomic nervous system are thought to contribute to changes in the complexity of the heart rate [89].

### 1.4 Spectral analysis of heart rate variability (HRV) and ageing

Following the pioneering work of Penaz et al. [1] and Hyndman et al. [2] on oscillatory processes in the ECG, Sayers [90] and Luczak and Lauring [91] studied rhythms in beat-to-beat heart rate signals. Akselrod et al. in 1981 [3] introduced spectral analysis of heart rate variability (HRV) as a non-invasive means of evaluating beat-to-beat cardiovascular control. In addition to the respiratory oscillations in HRV around 0.3 Hz at what were called high frequencies (HF), spectral peaks were reported at low frequencies (LF) around 0.1 Hz, and at very low frequencies (VLF) below 0.05 Hz [2,3]; this work was based on relatively short-term recordings. Ultra-low frequency (UFL) components were later observed in 24 h long-term recordings [92]. Many studies have investigated how sympathetic and parasympathetic activities affect these components. HF is considered to represent vagal control of the heart rate and LF contains contributions from both the vagal and sympathetic nervous systems. The ratio LF/HF is regarded by many researchers as a measure of sympathovagal balance [93].

However, the majority of these studies were done using FFT and autoregressive (AR) spectral estimation [92]. By these methods, frequencies below 0.05 Hz could not be studied (see above). To overcome this deficiency, Lotrič et al. [94] used the wavelet transform for spectral analysis, enabling them to study age-related spectral changes in the range 0.0095–0.6 Hz. In what follows, we discuss an additional frequency interval, 0.005–0.0095 Hz, and we also consider gender differences, which were not mentioned by Lotrič et al., as well as ageing.

### 1.5 Structural and functional changes in the cardiovascular system with age

Cardiovascular structure and function change with age, affecting the function of the heart and other organs, and perhaps causing diseases.

One of the major changes with ageing is the remodelling of large arteries, when there is an increase in wall thickness and enlargement of the lumen. Arterial stiffening is another hallmark of arterial ageing [95]. The geometry and diastolic function of the left ventriculum alter substantially with age [96].

Also associated with ageing, there are alterations in the function of the endothelium, the thin layer of cells that line the inner surfaces of all blood vessels. Endothelial control of vasomotor tone changes with age and the alteration impairs vascular adaptation to variations in flow, especially those induced by exercise and ischaemia. The endothelium normally releases vasoactive substances, such as nitric oxide (NO), but its ability to do so also changes with age. An impairment of endothelial-dependent relaxation, which is mediated especially by NO, is observed in aged subjects. Most studies indicate that ageing is associated with a decrease in NO production and release [97].

### 1.6 Blood flow with iontophoresis and ageing

The endothelium was once thought to serve just as passive lining for the blood vessels. However Furchgott and Zawadzki 1980 [98] found that the relaxation evoked by acetylcholine in the rabbit aorta is mediated only in the presence of the endothelium, and numerous later studies have confirmed that the endothelium plays an important role in regulating local vascular tone. It does so by releasing vasodilating and vasoconstricting substances.

Iontophoresis is a technique that allows for transdermal delivery of polar drugs though the skin by passing a small current. Here, we are especially interested in delivering the vasoactive endothelial-dependent and endothelial-independent substances acetylcholine (ACh) and sodium nitroprusside (SNP) respectively. Details are provided in Appendix B.2.3. Iontophoresis has been widely used to assess how endothelial vasodilation changes with essential hypertension, heart failure, arteriosclerosis and exercise, as well as ageing. Blood flow was measured by using laser Doppler flowmetry (LDF) at sites into which ACh and SNP were delivered by iontophoresis and then the blood flow signals were analysed by means of a wavelet transform according to [99]. This is a non-invasive measurement that enables one to acquire data to assess the state of the human cardiovascular system *in vivo*. It has been especially useful in identifying the physiological origins of the several spectral peaks revealed in earlier studies [99–104], such as the endothelial, neurogenic and myogenic, as well the respiratory and cardiac components [105]. The combination of iontophoresis and wavelet analysis allows endothelial function to be compared between subjects of different ages by focusing on the frequency interval(s) corresponding to endothelial activity.

Earlier ageing studies of blood flow based on iontophoresis have in some cases reported that endothelial-dependent vasodilation decreased with increasing age [47,48]. There are also some studies in which gender differences in endothelial-dependent vasodilation were observed [106,107]. But wavelet analysis was not used, and neither were the relevant oscillatory components examined individually. Here we review LDF measurement of blood flow combined with both iontophoresis and wavelet analysis and we show that this combination is very revealing.

## 2 Instantaneous frequency and phase

### 2.1 Description of the phase dynamics

In this section, we review briefly the phase dynamics approach to coupled oscillatory systems, following Kuramoto [24]. Phase dynamics provides a way of describing a system with only one variable, the phase. We first explain how the phase is defined and how its dynamics is obtained by use of one of the reduction methods that will be explained below in more detail.

#### 2.1.1 Small perturbations in general

Suppose that X(t) develops its dynamics according to dX/dt=F(X) and that there is a linearly stable T-periodic solution $X_{0}$ which satisfies

(1) $$\frac{dX_{0}}{dt}=F\left(X_{0}\right)\text{,}\;X_{0}\left(t+T\right)=X_{0}\left(t\right)\text{.}$$

Let C denote a closed orbit corresponding to $X_{0}$. Clearly the phase ϕ can be defined on C as a variable linearly increasing with time, as follows:

(2) $$\frac{d\phi \left(X\right)}{dt}=\omega \text{,}\;\omega =\frac{2\pi }{T},\;X\in C\text{.}$$

Now let us add a small perturbation p(t) to the dynamics. At this stage, p(t) can be anything. It may depend on the variable X or on the variables of other oscillators. The dynamics of X can then be expressed in the following equation:

(3) $$\frac{dX}{dt}=F\left(X\right)+p\left(t\right)\text{.}$$

**Fig. 1:**
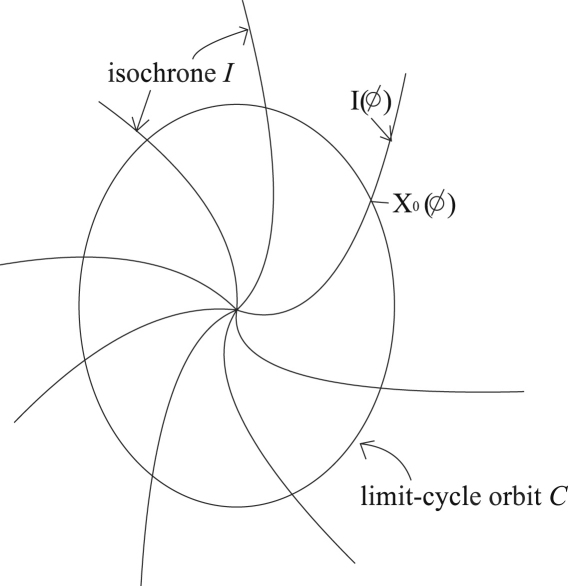
Explanation of the isochrone. The closed curve represents the limit cycle orbit C. Curves on which all the points have the same phase are called isochrones, I(ϕ). A crossing point of C and I(ϕ) is written as $X_{0}\left(\phi \right)$. The centre of the limit cycle C, where all the isochrones meet, is a singular point where the phase cannot be defined.

Once the perturbation has been added, the orbit does not correspond exactly to C, but is expected to be a bit away from C. Consequently, the phase needs to be defined, not only on C, but also throughout the region close to C: the definition can be extended to the region G containing the neighbourhood of C in the case of the dynamical system dX/dt=F(X). All paths starting from I(ϕ) approach the point starting from $X_{0}\left(\phi \right)$ on C, the crossing point of C and I(ϕ) (shown in Fig. 1 as t→∞). This means that the phase of the same isochrone remains the same. The above equation then leads to

(4) $$\left({\mathrm{grad}}_{X}\phi ,F\left(X\right)\right)=\omega \text{,}$$

where (a,b) represents the inner product of vectors a and b.

Note that the definition of phase is made for the perturbation-free system, but it can also be applied to the system in the presence of the perturbation.

On introducing this phase variable, the dynamics in the region G is finally described as

(5) $$\frac{d\phi \left(X\right)}{dt}=\left({\mathrm{grad}}_{X}\phi ,F\left(X\right)+p\left(t\right)\right)=\omega +\left({\mathrm{grad}}_{X}\phi ,p\left(t\right)\right)\text{.}$$

It should be noted that ${\mathrm{grad}}_{X}\phi$ on the right hand side is a function of position X, and that Eq. (5) is not a closed equation for the phase ϕ. However, if the perturbation is small, the value can be calculated approximately from the value on C as

(6) $$U^{*}\left(\phi \right)\equiv {\mathrm{grad}}_{X}\phi {|}_{X_{0}\left(\phi \right)}\text{.}$$

By use of this $U^{*}$, the phase equation under perturbation p(t) can be obtained as

(7) $$\frac{d\phi }{dt}=\omega +\left(U^{*}\left(\phi \right),p\left(t\right)\right)\text{.}$$

If the perturbation is given and it is a function of ϕ, Eq. (7) can be closed in terms of ϕ. We now consider an example.

#### 2.1.2 Small deviation from the original dynamical system

In this subsection, we discuss the case where the dynamical equation deviates from F(X) to F(X)+δF(X). In this case, p(t)=δF(X) and Eq. (7) becomes

(8) $$\frac{d\phi }{dt}=\omega +\left(U^{*}\left(\phi \right),\delta F\left(X\right)\right)\text{.}$$

In the first approximation, δF(X) can be replaced by $\delta F\left(X_{0}\left(\phi \right)\right)$. Then Eq. (8) becomes

(9) $$\frac{d\phi }{dt}=\omega +\left(U^{*}\left(\phi \right),\delta F\left(X_{0}\left(\phi \right)\right)\right)\text{.}$$

This is closed for ϕ. An important operation called averaging is implemented in the next step by introduction of a new variable ψ as

(10) ϕ=ωt+ψ.

Without the perturbation, ψ is a variable independent of time and it represents the initial phase, but under small perturbation it is a variable that changes slowly with time. The dynamics of ψ becomes

(11) $$\frac{d\psi }{dt}=\left(U^{*}\left(\omega t+\psi \right),\delta F\left(X_{0}\left(\omega t+\psi \right)\right)\right)\text{.}$$

Because its dynamics is very slow, ψ can be considered as approximately constant during one period 2π/T. In fact, ψ is so slow compared to ωt that it is expected that the averaging of the right hand side occurs on the time scale of ψ. The dynamics of ψ can thus be expressed as

(12) $$\frac{d\psi }{dt}=\delta \omega \text{,}$$

(13) $$\delta \omega \equiv \frac{1}{2\pi }\int_{0}^{2\pi }d\theta \left(U^{*}\left(\theta +\psi \right),\delta F\left(X_{0}\left(\theta +\psi \right)\right)\right)\text{.}$$

It should be noted that δω is not dependent on ψ, but constant, since the integrated function in the right hand side is a 2π-periodic function. The equation

(14) $$\frac{d\phi }{dt}=\omega +\delta \omega$$

indicates that the deviation of the original dynamical system leads to a deviation of the frequency in the phase dynamics, i.e. frequency modulation.

### 2.2 Analytic methods for detecting instantaneous phase

In analysing cardiovascular (and many other) signals, the first thing that we have to do is to define their phases quantitatively. There are three methods for defining instantaneous phase. They are based respectively on peak detection, the Hilbert transform, and the wavelet transform. The first method can be used to study entrainment directly [108] or to obtain instantaneous phase [109]. The second method was originally introduced by Gabor [110] and brought into the context of synchronization by Rosenblum et al. [111]. The third wavelet-based method was introduced by Lachaux et al. [112] and independently by Bandrivskyy et al. [113]. Wavelet analysis is explained in Section 4.2. Phase synchronization between EEG signals from the right and left hemispheres of a rat’s brain was investigated by use of both the Hilbert and wavelet transforms by Quiroga et al. [114] who found that they obtained similar results by the two methods.

#### 2.2.1 Marked events

If each cycle of a signal contains distinctive events that can be marked to characterize the oscillator, the phase can be defined by using the times of these events,

(15) $$\phi \left(t\right)=2\pi \frac{t-t_{k}}{t_{k+1}-t_{k}}+2\pi k\text{,}\;t_{k}<t<t_{k+1}\text{,}$$

where $t_{k}$ and $t_{k+1}$ are the times of the kth and (k+1)th marked events. By this definition, the phase increases linearly with time. It should be noted that this method corresponds to phase definition via Poincaré section [109]. In some cases, we can find a projection of an orbit on a plane (x,y) that rotates around a point $\left(x_{0},y_{0}\right)$. We can choose a Poincaré section, and $t_{k}$ is then the time of the kth crossing of the Poincaré surface. In our case, the Poincaré section will be defined by the plane of y=max.

#### 2.2.2 The Hilbert transform

When a signal g(t) is obtained, there is a way to establish its amplitude and phase by constructing the so-called analytic signal ζ(t) from the original signal g(t), according to the equation

(16) $$\zeta \left(t\right)=g\left(t\right)+ıg_{H}\left(t\right)=A\left(t\right)e^{ı\phi \left(t\right)}\text{,}$$

where $g_{H}\left(t\right)$ is the Hilbert transform of g(t) written as

(17) $$g_{H}\left(t\right)=\pi^{-1}\mathrm{PV}\int_{-\infty }^{\infty }\frac{g\left(\tau \right)}{t-\tau }d\tau \text{.}$$

Here PV means evaluation of the integral in the sense of the Cauchy principal value. The instantaneous amplitude A(t) and phase ϕ(t) are determined by Eq. (16). Then the phase can be calculated as

(18) $$\phi \left(t\right)=\arctan\frac{g_{H}\left(t\right)}{g\left(t\right)}\text{.}$$

Note that the phase obtained by this method ranges from −π to π.

From Eq. (17), it can be seen that the Hilbert transform is the convolution of the functions g(t) and 1/πt. According to a property of convolution, the Fourier transform ${\overset{ˆ}{g}}_{H}$ of $g_{H}\left(t\right)$ is the product of the Fourier transform of g(t) and 1/πt. For physically relevant Fourier frequencies f>0, ${\overset{ˆ}{g}}_{H}\left(f\right)=-ı\overset{ˆ}{g}\left(f\right)$, which means that the Hilbert transform can be seen as a filter whose amplitude response is unity and whose phase response is a π/2 lag at all frequencies.

It should be remarked that this method is reasonable only when the original signal g(t) is a narrow band one. Real signals usually contain a wide range of frequencies because of noise or other factors, and some filtering may be necessary in order to use this method.

### 2.3 Application to cardiovascular signals

#### 2.3.1 Heart rate variability (HRV) and respiratory frequency variability (RFV)

The instantaneous frequencies can be introduced by using phase information obtained according to the methods described above. If the phase reaches 2π for the kth and (k+1)th time at $t_{k}$ and $t_{k+1}$ respectively, the instantaneous frequency $f_{i}$ is defined as

(19) $$f_{i}\left(t_{k,k+1}\right)=\frac{1}{\left(t_{k+1}-t_{k}\right)}\;\mathrm{where}\;t_{k,k+1}=\frac{\left(t_{k}+t_{k+1}\right)}{2}\text{.}$$

The instantaneous frequency between $t_{k,k+1}$ and $t_{k+1,k+2}$ is defined by linear interpolation as

(20) $$f_{i}\left(t\right)=\frac{f\left(t_{k+1,k+2}\right)-f\left(t_{k,k+1}\right)}{t_{k+1,k+2}-t_{k,k+1}}\left(t-t_{k,k+1}\right)+f_{i}\left(t_{k,k+1}\right)\text{,}\;t_{k,k+1}<t<t_{k+1,k+2}\text{.}$$

**Fig. 2:**
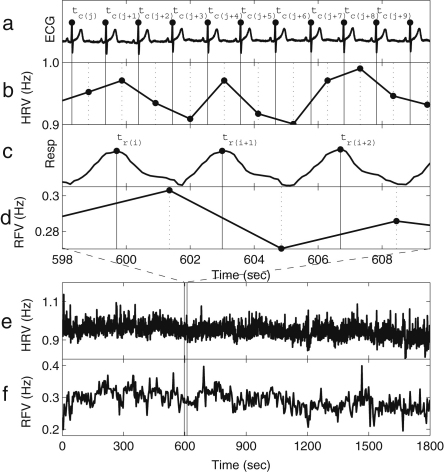
Cardiovascular signals. (a) ECG signal during a time segment where R-peaks are marked at times $t_{c\left(j+k\right)}$ for k=0,…,9. (b) The instantaneous frequency $1/\left(t_{c\left(j+k+1\right)}-t_{c\left(j+k\right)}\right)$ marked at times $\left(t_{c\left(j+k\right)}+t_{c\left(j+k+1\right)}\right)/2$ for k=0,…,9 forms the HRV signal (a time series of instantaneous HR). (c) The respiratory signal during the same time segment where maxima are marked at times $t_{r\left(i+l\right)}$ for l=0,1,2. (d) The instantaneous frequency $1/\left(t_{c\left(i+l+1\right)}-t_{c\left(i+l\right)}\right)$ marked at times $\left(t_{c\left(i+l\right)}+t_{r\left(i+l+1\right)}\right)/2$ for k=0,1,2 forms the RFV signal (a time series of instantaneous RF) during the time segment. (e) The HRV signal and (f) the RFV signal during the whole measurement period. The averages of (instantaneous) HR and (instantaneous) RF are time-averaged HRV and RFV over the whole time series, and the standard deviations of (instantaneous) HR and (instantaneous) RF are the standard deviations of the HRV and RFV time series, respectively.

This method is applied to an individual signal, e.g. to either or both of the ECG and respiratory signals. The first part of the analysis is to determine the heart rate (HR) and the respiratory frequency (RF). Their instantaneous frequencies as functions of time are then heart rate variability (HRV) and respiratory frequency variability (RFV), respectively. HR and RF are normally used to represent time-averaged values or values at one instant, rather than as functions of time. HRV is a well-established indicator for cardiac regulation. The existence of fluctuations in heart rate was noticed as early as 1733 by Hales [115], related to the respiratory oscillation. This modulation is known as respiratory sinus arrhythmia (RSA). RSA has sometimes been assessed regardless of any distinction of frequency interval within HRV, for example by using RSA curves [116,117], and sometimes assessed using the spectral power corresponding to the frequency interval of respiration [118]. In our work, we use the latter method for assessing RSA. Actual examples of HRV and RFV signals are shown in Fig. 2, where the RSA can be seen. These values relate to single periods during which the phase starts from zero and resets to 2π. As explained in Section 2.1.2, the variation of the frequency of HRV and RFV can be considered to come from the term of $\left(U^{*}\left(\phi \right),p\left(t\right)\right)$ in Eq. (7), where the perturbation p(t) can be the parameter change described in Section 2.1.2 and the coupling to other oscillators such as the respiratory oscillation as we discuss below in Section 5.1. The respiratory oscillation evidently has an especially important role in modulating the heart rate, given that HRV contains an oscillatory component corresponding to respiration [5]. The spectral analysis of HRV will be discussed in Section 4.3.1, where the origin of the other oscillatory processes modulating the heart rate will also be discussed.

#### 2.3.2 Effects of ageing on heart rate (HR) and respiration frequency (RF)

**Fig. 3:**
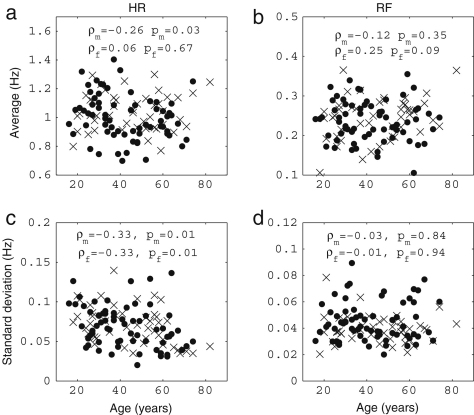
The effect of ageing on (a) average (instantaneous) HR, (b) average RF, (c) the standard deviation of (instantaneous) HR and (d) the standard deviation of (instantaneous) RF. The values of $\rho_{m}$ and $p_{m}$ represent correlations with age and the probability for males, and the values of $\rho_{f}$ and $p_{f}$ represent those for females. The filled circles represent males, and the crosses represent females.

It is well known that the standard deviation of (instantaneous) HR decreases significantly with age [38,39,41,120]. In Fig. 3, we present the results of 30 min recordings conducted according to the procedures of Appendix B. The method used for calculating the average and standard deviations of HR and RF is described in the caption of Fig. 2: we calculated Spearman’s rank correlation coefficients and their statistical significance using the method described in Appendix C.2.2, implemented in MatLab (MatWork). Since Spearman’s rank correlation coefficient does not suppose a particular relationship between two variables, we mostly do not plot any fitted curves but just give the correlation coefficients. We denote the Spearman’s rank correlation coefficient by ρ, the linear correlation coefficient by r and the significance probability for each by p. There is significant correlation with age in the standard deviation of (instantaneous) HR both for males (ρ=−0.33,p=0.01) and for females (ρ=−0.33,p=0.01), where by a significant correlation we mean p≤0.05. The other values, average (instantaneous) HR, average (instantaneous) RF and the standard deviation of (instantaneous) RF do not show significant correlation with age for either males or females. Next, we compared the differences between males and females in each age group by using the Wilcoxon rank sum test, which is discussed in Appendix C.1.2. Note that this test considers only the ranks of the two groups, and not their absolute values, which means that the significance test is not much affected if outliers raise the standard deviations. The average (instantaneous) RF of females is significantly higher than that of males in the aged population above 55 years (p=0.05, male 0.22±0.08, female 0.27±0.04), whereas the other values do not show significant gender differences. Throughout this review, all correlations with age were quantified using the Spearman’s rank correlation coefficients and all comparisons between two groups based on our data were conducted using the Wilcoxon rank sum test.

**Fig. 4:**
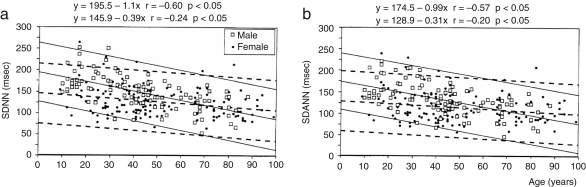
Relationships between age and HRV determined through the standard deviation of all normal sinus RR intervals over 24 h (SDNN) in (a) and the standard deviation of the averaged normal sinus RR intervals for all 5 min segments (SDANN) in (b) for healthy male (open squares) and female (solid circles) subjects. The fitted regression line and upper and lower 95% confidence limits are shown by the full lines for male subjects and the dashed ones for females. The figure is taken from the paper by Umetani *et at* [119] reporting measurements on 112 males and 148 females ranging in age from 10 to 99 years.

The significant decrease in the standard deviation of (instantaneous) HR mentioned above was reported, not only for 30 min recordings, but also for 24 h ones, enabled by a recent development in measurement technology, the Holter monitor. Even for these longer recordings, the trend still holds, as shown in Fig. 4 by Umetani et al. [119].

There are, however, some differences in the results reported by different authors. For example, Stein et al. [120] reported that there is a significant decrease in average heart rate for males, whereas Umetani et al. [119] reported that the significant decrease in heart rate is only for females. Ryan et al. [38] reported that average heart rate did not change between young and aged groups, as we observed. Umetani et al. [119] observed that the standard deviation was lower in female than male subjects, which we did not observe. The difference probably comes from the difference of recording time, the number of subjects and the lifestyles of the subjects. Note also that 24 h recordings contain the effects and artefacts resulting from the subjects’ day-to-day lives, whereas 30 min recordings are made for subjects that are relaxed and lying on a bed. There is a report by Dietrich et al. that lifestyle factors such as exercise, alcohol and smoking affect HRV. Nonetheless, the decrease of HRV (the decrease of the standard deviation of HR) with age seems to be robust.

## 3 Complexity analysis

### 3.1 Overview of existing results

In this section, we overview results obtained before the introduction of detrended fluctuation analysis (DFA) and detrended moving analysis (DMA). The history of development in these latter areas has already been described above in Section 1.3.

The complexity is independent of the mean and variance of a signal, and special techniques are required for its determination. Note e.g. that two sine waves of different amplitude can be thought to have the same complexity, although they have different variances.

Chaos theory provides meaningful ways of quantifying complexity. One is the dimension, which is interpreted as the number of variables in the difference or differential equations needed to construct a dynamical system that will reproduce the measured signals. Another is the entropy, which is related to the amount of information needed to predict the future state of the system. A larger dimension or larger entropy implies greater complexity [8,77]. When the approximate dimension and entropy *ApEn* were calculated in the signals of the blood pressure and heart rate [41], it was observed that younger subjects have higher complexity than older subjects in all cases: for both blood pressure and HRV, and for the both measures. Ryan et al. calculated *ApEn* and reported that the complexity of heart rate dynamics is higher in women than in men [38]. Higuchi suggested quantification of the complexity based on the fractal dimension [121,122]. By this method, fractal changes in heart rate with ageing and heart failure were studied [123]. The relationship between complexity and ageing has been reviewed by Lipsitz et al. [42].

Most of the signals or time series measured from physical, biological, physiological and economic systems are non-stationary in character and exhibit complex self-similar fluctuations over a broad range of space or time scales. To see the scaling property, a time series is expected to grow with the window size in a power-law way, and to be unbounded. But a real signal is inevitably bounded. The trick for solving this paradox is to integrate the signals. The integration of a signal is the critical first step common to all the methods used to calculate the complexity. Starting with an original signal g(i), where i=1,…,N, and N is the length of the signal, the first step of the Hurst exponent, DFA, and DMA methods is to integrate g(i) and obtain the integrated signal y(i) as

(21) $$y\left(i\right)=\overset{i}{\underset{j=1}{\sum }}\left[g\left(j\right)-\overset{¯}{g}\right]\text{,}$$

where

(22) $$\overset{¯}{g}\equiv \frac{1}{N}\overset{N}{\underset{j=1}{\sum }}g\left(j\right)\text{.}$$

To calculate the Hurst exponent, we have to calculate the standard deviation,

(23) $$S\left(N\right)={\left[\frac{1}{N}\overset{N}{\underset{t=1}{\sum }}{\left\{g\left(i\right)-\overset{¯}{g}\right\}}^{2}\right]}^{1/2}\text{,}$$

and the range,

(24) $$R\left(N\right)=\underset{1\leq i\leq N}{\max}y\left(i\right)-\underset{1\leq i\leq N}{\min}y\left(i\right)\text{.}$$

The Hurst exponent H is then defined as

(25) $$R/S={\left(cN\right)}^{H}\text{,}$$

where the coefficient c was taken as 0.5 by Hurst. He found that the ratio R/S is very well described for a large number of natural phenomena by the above empirical relation. The relation between the Hurst exponent and the fractal dimension is simply D=2−H.

A Hurst exponent of 0.5<H<1 represents persistent behaviour. Persistence means that if the curve has been increasing for a period, it is expected to continue for another period. A Hurst exponent of 0<H<0.5 shows anti-persistent behaviour. After a period of decreases, a period of increases tends to occur.

### 3.2 Analytical methods: detrended moving analysis (DMA) and detrended fluctuation analysis (DFA)

The DFA method is a modified root mean square (rms) analysis of a random walk. Both the DMA and DFA methods are based on the fractal property. Following [79,80], we summarize below the procedures for implementation.

A time series is self-similar if it satisfies

(26) $$y\left(i\right)\equiv a^{\alpha }y\left(\frac{i}{a}\right)\text{,}$$

where y(i) is the integrated original signal g(i), and ≡ means that the statistical properties of the two sides of the equation are identical (the two sides have the identical probability distribution as a properly rescaled process). The x-axis is rescaled as t→t/a and the y-axis as $y\to a^{\alpha }y$.

Suppose that the original signal length is $n_{2}$, and that a window of length $n_{1}<n_{2}$ is taken to test for self-similarity compared to the original signal. Then the magnification factor of the x-axis, a, is $n_{2}/n_{1}$. Suppose that the probability distribution is $s_{2}$ for the original signal and $s_{1}$ after magnification. Then the magnification factor of the y-axis $a^{\alpha }$ is $s_{2}/s_{1}$. The self-similarity parameter α is expressed as

(27) $$\alpha =\frac{\ln M_{x}}{\ln M_{y}}=\frac{\ln s_{2}-\ln s_{1}}{\ln n_{2}-\ln n_{1}}\text{.}$$

To calculate s, the DFA method uses filtering by polynomial functions. At first, the integrated signal y(i) is divided into boxes of equal length n. In each box, we fit y(i) using a polynomial function $y_{n}\left(i\right)$, which represents the local trend in that box. When an lth-order polynomial function is used for filtering, we call the method DFA-l.

Next, the integrated profile y(i) is detrended by subtracting the local trend $y_{n}\left(i\right)$ in each box of length n and we can get $Y_{n}$ as

(28) $$Y_{n}\left(i\right)\equiv y\left(i\right)-y_{n}\left(i\right)\text{.}$$

By this procedure, non-stationarity in the form of polynomial trends is eliminated.

Finally, for each box, the rms fluctuation of the integrated and detrended signal is defined as

(29) $$F\left(n\right)\equiv \sqrt{\frac{1}{N}\overset{N}{\underset{i=1}{\sum }}{\left[Y_{n}\left(i\right)\right]}^{2}}$$

and F(n) is then considered as s in the above discussion.

The DMA method uses a moving average method to get $y_{n}$. For example, the simple backward moving average is

(30) $$y_{n}\left(i\right)\equiv \frac{1}{n}\overset{n-1}{\underset{k=0}{\sum }}y\left(i-k\right)\text{.}$$

For further details, see [124]. Then we subtract the trend $y_{n}$ from the original signal as in Eq. (28). We can calculate F(n) in the same way.

The calculation of F(n) is made for varied box lengths n to obtain a power-law relationship between the rms fluctuation function F(n) and the scale n in the form of

(31) $$F\left(n\right)∼n^{\alpha }\text{.}$$

A linear relationship between log(F(n)) and log(n) indicates the presence of scaling (self-similarity). The fluctuations in the small boxes are related to those in the larger boxes in a power-law fashion. The slope of the graph between log(F(n)) and log(n) determines the self-similarity parameter α, which quantifies the presence or absence of fractal correlation properties in the signals. For example, 1/f noise with long range correlation returns α≃1.0, white noise with uncorrelated randomness returns α≃0.5 and Brown noise returns α≃1.5.

Because power laws are scale invariant, F(n) is also called the scaling function and the parameter α is the scaling exponent.

These two methods are both suitable for non-stationary signals such as the physiological signals described above. Which method is better, DMA or DFA? There has been a comparative study of the performance of DFA and DMA methods [124]. It investigated how accurately these methods reproduce the exponent α, and the limitations of the methods when applied to signals with small or large values of α. It was shown [124] that DMA tends to underestimate the exponent if it is larger than unity whereas the DFA, especially DFA-1, shows relatively good correspondence to the true values over a wide range of α. In our study, the exponents went beyond unity and therefore we adopted the DFA-1 approach.

### 3.3 The relationship between the exponents obtained by DFA and from the auto-correlation function

**Fig. 5:**
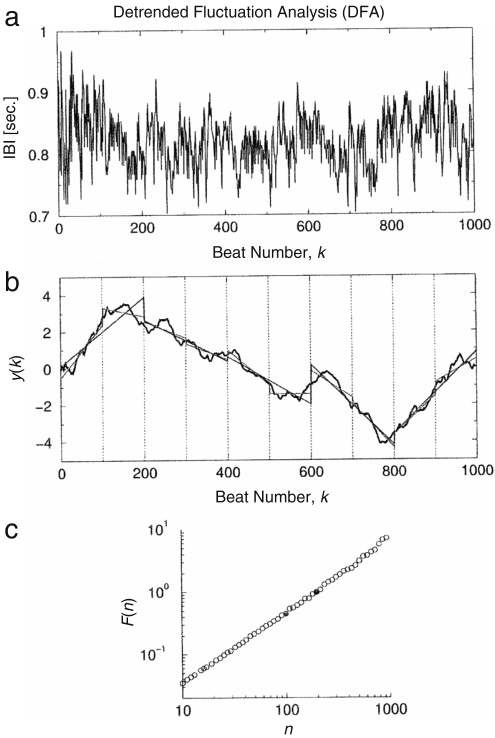
Illustration of how the DFA algorithm is used to test for scale invariance and long range correlations. (a) Interbeat interval (IBI) time series (RR intervals, in seconds) from a healthy young adult. (b) The full black curve is the integrated time series, y(k). The vertical dotted lines indicate boxes of size n−100 beats. The straight line segments represent the trends estimated in boxes of size n=100 and 200 beats by linear least-square fits. (c) The rms deviations, F(n), in B are plotted against the box size n, in a double-logarithmic plot. The two filled circles are the data points for F(100) and F(200). The straight line graph indicates power-law scaling.

Many simple systems have an auto-correlation function that decays exponentially with time. However it was discovered that in a system composed of many interacting subsystems, it decays not exponentially but in a power-law form [19,125]. This implies that there is no single characteristic time in a complex system. If correlations decay in a power-law manner, the system is called *scale-free* because there is no characteristic scale associated with a power law. Because at large time scales a decreasing power law gives larger values than a decaying exponential function, correlations described by power laws are termed “long range correlations” in the sense that they are of larger range than an exponentially decaying function. The DFA method can detect such long range correlations, as illustrated in Fig. 5, and here we will discuss the relationship between the exponent and the correlation function.

The exponent α (self-similarity parameter) which is calculated from an integrated time series is related to the more familiar auto-correlation function, C(τ), or the Fourier spectrum, S(f), of the original (non-integrated) signal. (It is well known that C(τ) and S(f) are related through the Fourier transform as $S\left(f\right)=\int_{-\infty }^{\infty }C\left(\tau \right)\exp\left(ı2\pi f\tau \right)d\tau$.)

White noise, whose value at each moment is completely uncorrelated with any previous value, has an auto-correlation function, C(τ), which is 0 for any non-zero τ (time lag). The exponent α of white noise is 0.5 [75].

An α greater than 0.5, and less than or equal to 1.0, indicates persistent long range power-law correlations, i.e., $C\left(\tau \right)∼\tau^{-\gamma }$. The relationship between α and γ is γ=2−2α. It should also be noted that the power spectrum, S(f), of the original (non-integrated) signal is also of a power-law form, i.e., $S\left(f\right)∼1/f^{\beta }$. Since the power spectral density is simply the Fourier transform of the autocorrelation function, β=1−γ=2α−1. The case of α=1 corresponds to 1/f noise (β=1).

When α<0.5, power-law anti-correlations are present such that large values are more likely to be followed by small values [126].

When α>1, correlations exist but cease to be of power-law form; α=1.5 indicates Brown noise, which is created by the integration of white noise. Unlike white noise, Brown noise is correlated because its instantaneous value depends on previous fluctuations and cannot stray too far from them in a short time. Brown noise has a spectral density proportional to $1/f^{2}$ and has stronger modulation in slow time scales.

The exponent α can also be viewed as an indicator of the roughness of the original time series: the larger the value of α, the smoother the time series. In this context, 1/f noise can be interpreted as a compromise between the complete unpredictability of white noise (a very rough form of noise) and the much smoother form of Brown noise [127].

### 3.4 Application to cardiovascular signals

#### 3.4.1 Application to HRV signals

As shown in Section 2.3.2, HRV exhibits a significant negative correlation with age. HRV has also been considered in terms of complexity analysis, yielding results that we review in this subsection.

**Fig. 6:**
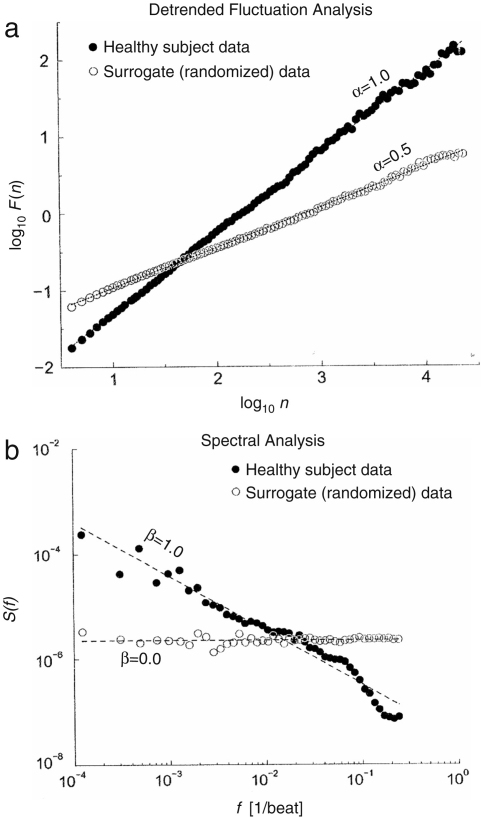
Fractal scaling analysis for 24 h interbeat interval time analysis. The filled circles represent data from a healthy subject, whereas the open circles are for an artificial time series (surrogate data) created by randomizing the sequential order of data points in the original time series. (a) Plot of log F(n) as a function of log(n) from DFA analysis. (b) Fourier spectral analysis. The spectra have been smoothed (binned) to reduce scatter. The observed α≃1.0,β≃1.0 for a healthy subject is consistent with 1/f noise. After randomization, α≃0.5,β≃0.0 is consistent with white noise.

**Fig. 7:**
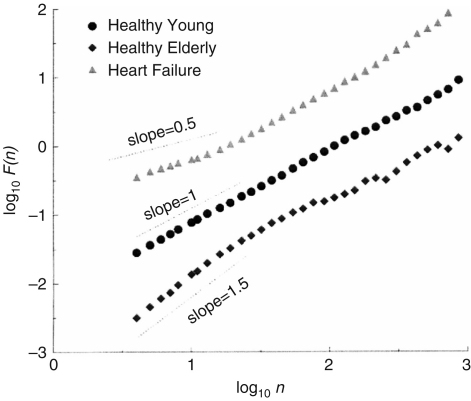
Scaling analyses of heartbeat time series in health, ageing and disease. Here, log F(n) is plotted as a function of log(n) for a healthy young subject, a healthy elderly subject, and a subject with congestive heart failure. To facilitate assessment of these scaling differences, the plots are vertically offset from each other.

We first discuss the results of Goldberger et al. [44]. They analysed interbeat intervals (the reciprocal of HRV), and reported that the result for a healthy subject is consistent with long range correlations (1/f noise). This was confirmed by an analysis of surrogate data, which revealed a loss of correlation properties as shown in Fig. 6. Further, it was reported by Peng et al. [128] that subjects with heart failure, and elderly subjects, show alterations in both short and long range correlation properties compared with healthy young subjects, as shown in Fig. 7. For example, the fluctuations of elderly subjects resembled Brown noise (α≃1.5) over a short range, whereas those of the heart failure patients resembled white noise (α≃0.5).

**Fig. 8:**
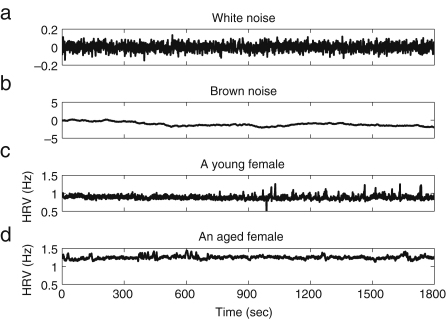
Time series of (a) white noise, band limited at 1  Hz, (b) its integrated time series (Brown noise), (c) HRV from a young female and (d) HRV from an aged female.

In order to check the robustness of our conclusions about the effect of ageing on complexity, we calculated the exponent α of signals analysed by use of the DFA method. Original HRV signals of a young and an old female, recorded by ourselves, are shown in Fig. 8, together with white and Brown noise signals for comparison. The HRV signals were determined from the intervals between R-peaks as explained in Section 2.3.1. The interval between successive R-peaks is usually around 1 s. According to Eq. (19), the sampling frequency of the HRV signal is by construction around 1 Hz although we made their effective sampling frequency 10 Hz by linear interpolation. To compare HRV signals to white noise and Brown noise, we generated and recorded white noise with a sampling frequency of 1 Hz and extended its sampling frequency to 10 Hz by linear interpolation, just as we did to the HRV signals. Then the Brown noise with sampling frequency 1 Hz was integrated from the band-limited white noise that we had generated, and its sampling frequency was also extended to 10 Hz by linear interpolation.

**Fig. 9:**
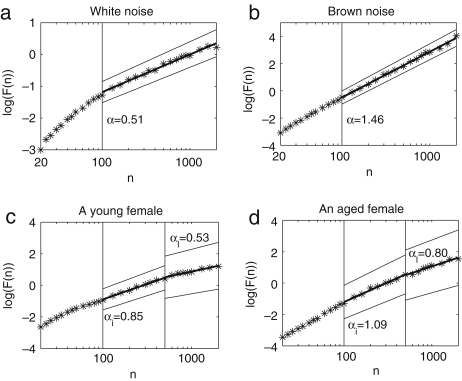
We show log–log plots of F(n) for (a) white noise, (b) Brown noise, (c) the HRV of a young female, and (d) the HRV of an aged female. In (a) and (b), the exponent α was calculated on the right side of the line, n=100. In (c) and (d), the exponent of the intermediate time scale $\alpha_{i}$ was calculated between the two lines (the intermediate range), n=100 and n=500, whereas the exponent of the long time scale $\alpha_{l}$ was calculated on the right side of the line n=500 (the long range). The fitted regression is shown by the solid line, and the upper and lower thinner lines represent 95% confidence intervals.

**Fig. 10:**
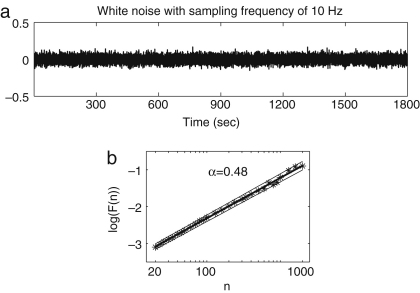
(a) Band-limited white noise whose effective sampling frequency has been increased to 10 Hz, and (b) the corresponding log–log plot of F as a function of n. The exponent can be defined uniquely from n=20 to n=2000 since we now have a sufficiently high sampling frequency: cf. Fig. 9a where two slopes are needed to obtain a satisfactory fit. The fitted regression is shown by the solid line, and the upper and lower thin lines represent 95% confidence intervals.

We take the number of points n between 20 and 2000, corresponding to 2–200 s. DFA results for white noise, Brown noise and the HRV signals from a young and an old female are shown in Fig. 9. When n is small, the slope between log(n) and log(F) deviates from 0.5 for white noise, as shown in Fig. 9a; this deviation is thought to be attributable to too small a sampling frequency. Fig. 10 shows the time series of the white noise with sampling frequency of 10 Hz without linear interpolation, and its DFA analysis, to compare with the band-limited white noise generated with a sampling frequency of 1 Hz and then converted to an effective sampling frequency of 10 Hz by linear interpolation. In Fig. 10, an exponent of ∼0.5 is obtained within the region from n=20 to n=200, and the information below n=100 does not have to be discarded as we did in the case of the band-limited white noise converted to an effective sampling frequency of 10 Hz by linear interpolation. For this reason, we relied on the result only when n is above 100, for all the other results, since they have the same sampling frequency as the band-limited white noise.

The exponent of the band-limited white noise is 0.51, which is close to the expected value of 0.5, as shown in Fig. 9a. We calculated the linear correlation coefficient and conducted a *runs test* as explained in Appendix C, in order to validate the linear regression. The coefficient r is 0.99 and the probability p is 0.0. The result of the runs test is that h=0 and p=1.0, which means that the null hypothesis that the distribution around the regression line is random cannot be rejected. The exponent of the Brown noise is 1.46, which is also close to the expected value of 1.50, as shown in Fig. 9b (the linear correlation: r=1.00 and p=0.0, the runs test h=0 and p=1.0). The exponents of human HRV signals cannot be determined uniquely, as in the case of white or Brown noise, because the slope between log(F) and log(n) changes depending on the size of n, as shown at the bottom of Fig. 9. We divided the n into two intervals so that the slope of HRV could be determined more reliably. The exponent $\alpha_{i}$ of intermediate time scale is defined by n within 100–500 (10–50 s) and the exponent $\alpha_{l}$ of long time scale is defined by 500<n<2000 (50–200 s). The physiological meaning of each interval will be described below in Section 4.3.1. For the HRV of a young female, $\alpha_{i}$ is 0.85 (the linear correlation: r=1.0 and p=0.0, the runs test h=0 and p=1.0) and $\alpha_{l}$ is 0.53 (the linear correlation: r=0.98 and p=0.0, the runs test h=0 and p=1.0). For the HRV of an aged female, the $\alpha_{i}$ is 1.09 (the linear correlation: r=0.99 and p=0.0, the runs test h=0 and p=1.0) and $\alpha_{l}$ is 0.80 (the linear correlation: r=0.99 and p=0.0, the runs test h=0 and p=1.0).

**Fig. 11:**
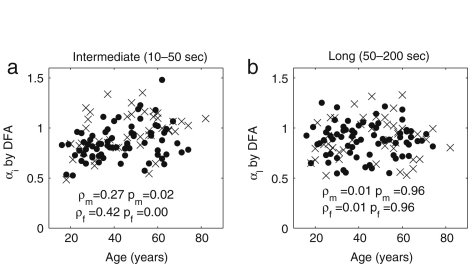
DFA correlation with age for (a) the exponent of intermediate range $\alpha_{i}$, and (b) the exponent of long range $\alpha_{l}$. The filled circles represent males, whereas the crosses represent females.

The results for all subjects are plotted separately for males and females in Fig. 11. The exponent $\alpha_{i}$ has significant correlation with age for both males (ρ=0.27,p=0.02) and females (ρ=0.42,p=0.00). There is no statistically significant gender difference in the younger age group below 40 years (p=0.19); for the older age group above 55 years there is a difference (p=0.06), but not one that is statistically significant.

In the long range (50–200 s), there is no significant correlation related to age for either males (ρ=0.01,p=0.96) or females (ρ=0.01,p=0.96). There is no significant gender difference, either: neither in the younger age group below 40 years (p=0.35), nor in the older age group above 55 years (p=0.80).

### 3.5 Discussion

There are several studies of ageing based on the use of DFA [44,49]. It was found that the DFA exponents increase with age, implying that complexity decreases with age. The exponents depend on the time window within which one performs the calculation. These authors took a size of 4 to 11 s for the short-term exponent and a size more than 11 s for the intermediate-term exponent. In our case, it was found out that the result below 10 s is not correct because of the lack of information in the original heart rate signals and we therefore discarded information below 10 s. And for reasons which we describe below in Section 4.3.1, we divided the window size into 10–50 s and 50–200 s. It should be noted that the choice of window size is of critical importance for getting correct results. Our results are consistent with the earlier result that the exponent increases with age when calculated on a time scale from 10 to 50 s. The HRV signals of younger subjects are relatively close to white noise, whereas those of aged subjects are relatively close to Brown noise in the intermediate time scale. That means that the HRV signals of aged subjects are less complex than those of young subjects. These results support the hypothesis that ageing has an associated loss of complexity [44]. The physiological origins of this decrease in complexity will be discussed in detail below, in Section 4.3.1, in relation to the detailed spectral analysis of HRV signals.

**Fig. 12:**
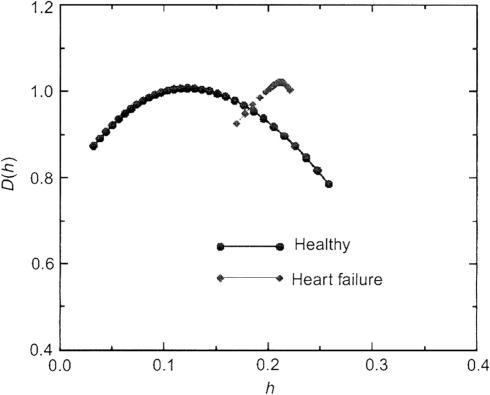
Singularity spectra of heart rate signals in health and disease. The function D(h) measures the fractal dimension of the subset that is characterized by a local Hurst exponent of value h. (The local Hurst exponent h is related to the exponent α of the DFA method by the relationship α=1+h.) Note the broad range in values of h with non-zero fractal dimension for the healthy heart beat, indicating multifractal dynamics. In contrast, data from a representative subject with severe heart failure show a much narrower range of h with non-zero fractal dimension, indicating a loss of multifractal complexity with a life threatening disease. The figure is taken from [16].

Although DFA is a good way to quantify the complexity, it has to be noted that it is intended only for monofractal signals, i.e. to measure only one exponent characterizing a given signal. It is reported that the heart rate data of healthy subjects are not monofractal but multifractal [44]. Different parts of the signal may have different scaling properties. Multifractal signals show self-similar (scale-invariant) fluctuation over a wide range of time scales, require a large number of indices to characterize their scaling properties, and are more complex than monofractal signals as shown in Fig. 12. For example, the slope between log(F(n)) and log(n) often changes dramatically around n=50 as can be seen in Fig. 9. This means that DFA is insufficient for identifying fractal correlation, and may in fact indicate the multifractal property.

In practice, it is not always clear how to choose the optimal window size within which to conduct the linear fitting, such that the size suits all subjects, whose signals may have significantly different characteristics. The more we divide a signal, the less information we obtain, because the number of points for the linear fitting decreases. However if we take too long a window, the assumption of a linear relationship between log(F(n)) and log(n) no longer holds. This is an inherent limitation of this method.

**Fig. 13:**
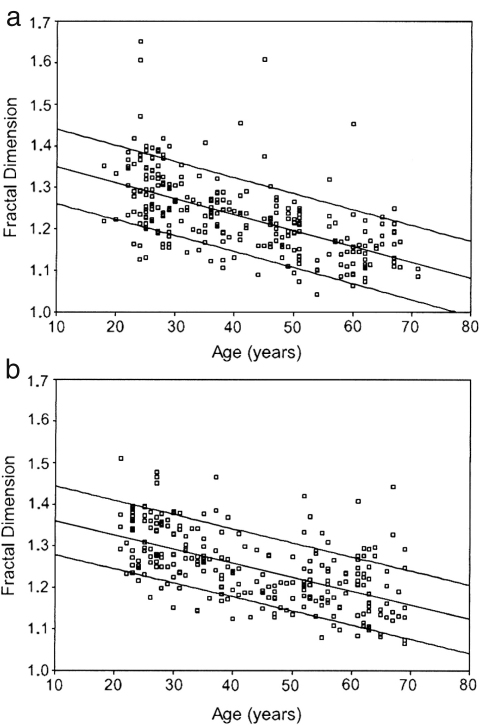
(a) Decrease of the fractal dimension (FD) of HRV with age in male subjects, r=−0.56(p<0.001), and (b) in female subjects, r=−0.56(p<0.001). The upper and lower lines represent the 90% confidence intervals. There is no significant difference in FD between male and female subjects, but the significant decrease with age persists regardless of whether the measurements were made during the day or night. A more detailed analysis using age intervals of 10 years showed a stabilization in the age decline of the FD at the age of 40 or more. The FD has correlation with other nonlinear indices such as *ApEn*, DFA exponents, and Lyapunov exponent. The figure is taken from [129].

Beckers et al. [129] reported that, during daytime hours, other nonlinear indices such as fractal dimension (FD) [130], correlation dimension (CD) [131], approximate entropy (*ApEn*) [132] and the Lyapunov exponent decrease with age for both males and females. Their FD results are shown in Fig. 13 as an example. They found in addition that the correlation with age in some indices disappeared during the night, especially for male subjects, i.e. there is day–night variation in the indices. It was also reported [129] that there is a tendency for higher nonlinearity during the night. The authors attribute the changes to vagal modulation of the heart rate.

Although more studies are needed to identify unambiguously the physiological reasons for the changes, it is noted that complexity is a useful measure of ageing or disease; it has yet to be established whether or not it can be used to discriminate between different diseases.

## 4 Detection of time-varying oscillatory components

In this section, we discuss the methods used to detect oscillatory components in the measured signals.

There are two major difficulties in the frequency analysis of cardiovascular signals. The first is the time-varying nature of the characteristic frequencies. As seen in the HRV and RFV of Fig. 2, the signals do not have a constant period, but their inherent cycles always fluctuate. The second problem is the broad frequency band within which the characteristic peaks are expected. There is always a problem of resolution in time and frequency, whatever method is used.

The FFT constitutes the basic method of frequency analysis, and it is still commonly used. But it has shortcomings when applied to the analysis of finite or non-stationary data. First of all, the FFT cannot follow a time-varying frequency. It produces only one picture of the frequency domain from a whole signal. If the signal has a time-varying frequency, the corresponding frequency peak is broadened. Furthermore, an abrupt change at any given instant affects the whole result. To overcome these drawbacks of the FFT, the short time Fourier transform was introduced by Gabor [110] in which a relatively narrow window is shifted along the signal to obtain information about the evolution with time, the FFT being performed within the window to obtain the current frequency components (see Section 4.1.1). But time and frequency resolution are dependent on the window length and the detection of low frequencies demands a wide window. Wavelet analysis is more suitable for signals with time-variable frequencies than Fourier analysis because a sudden change has a less global effect. This is a significant merit, because a single movement of the body during measurement could easily destroy the entire analysis in the case of FFT. Moreover, it is more accurate with low frequencies because it is a scale-independent method in terms of frequency (see Section 4.2).

### 4.1 Analytical methods

#### 4.1.1 Fourier analysis

The Fourier transform method is one which detects the frequency components in a time-domain signal g(u) by use of the following equation:

(32) $$\overset{ˆ}{g}\left(f\right)=\int_{-\infty }^{\infty }g\left(u\right)e^{-2\pi ıt}du\text{.}$$

The original signal can be recovered by using the inverse Fourier transform,

(33) $$g\left(u\right)=\int_{-\infty }^{\infty }\overset{ˆ}{g}\left(f\right)e^{2\pi ıt}df\text{.}$$

The energy of the signal is defined as

(34) $$E_{tot}=∥g{∥}^{2}=\int_{-\infty }^{\infty }{\left|g\left(u\right)\right|}^{2}du\text{.}$$

The total energy in the frequency domain is defined as

(35) $$∥\overset{ˆ}{g}{∥}^{2}=\int_{-\infty }^{\infty }{\left|\overset{ˆ}{g}\left(f\right)\right|}^{2}df\text{.}$$

Plancherel’s theorem, which is equivalent to Parseval’s theorem for Fourier analysis, states that

(36) $$∥g{∥}^{2}=∥\overset{ˆ}{g}{∥}^{2}\text{.}$$

#### 4.1.2 The short time Fourier transform

The Fourier transform cannot deal with properties that are local in time. To overcome this problem, the short time Fourier transform (STFT) was introduced. A window w(u) of fixed length, commonly a Hann or Gaussian function centred around zero, is shifted along in time t to obtain the local information around t. Information about the original signal g(u) in the time–frequency domain $\overset{ˆ}{g}\left(f,t\right)$ is then obtained from

(37) $$\overset{ˆ}{g}\left(f,t\right)=\int_{-\infty }^{\infty }w\left(u-t\right)g\left(u\right)e^{-2\pi ıt}du\text{.}$$

The original signal is reconstructed as

(38) $$g\left(u\right)=\frac{1}{2\pi \left\|w^{2}\right\|}\int_{-\infty }^{\infty }dt\int_{-\infty }^{\infty }\overset{ˆ}{g}\left(f,t\right)w\left(u-t\right)e^{2\pi ıt}df\text{.}$$

In analogy to Plancherel’s theorem, the energy is expressed as

(39) $${\left\|g\right\|}^{2}=\int_{-\infty }^{\infty }{\left|g\left(u\right)\right|}^{2}du=\frac{1}{\left\|w^{2}\right\|}\int_{-\infty }^{\infty }\int_{-\infty }^{\infty }{\left|\overset{ˆ}{g}\left(f,t\right)\right|}^{2}dfdt\text{,}$$

where $\left\|w^{2}\right\|=\int_{-\infty }^{\infty }{\left|w\left(t\right)\right|}^{2}dt$.

The uncertainty principle can be used here to emphasize that accuracy of localization in time, and frequency resolution, cannot be optimized simultaneously;

(40) $$t_{*}\equiv \frac{1}{\left\|w\right\|}\int_{-\infty }^{\infty }{\left|w\left(t\right)\right|}^{2}tdt\text{,}$$

(41) $$f_{*}\equiv \frac{1}{\left\|\overset{ˆ}{w}\right\|}\int_{-\infty }^{\infty }{\left|\overset{ˆ}{w}\left(f\right)\right|}^{2}f\;df\text{,}$$

where $\left\|{\overset{ˆ}{w}}^{2}\right\|=\int_{-\infty }^{\infty }{\left|\overset{ˆ}{w}\left(t\right)\right|}^{2}df$. $\Delta_{t}$ and $\Delta_{f}$ are determined by

(42) $${\Delta_{t}}^{2}\equiv \frac{1}{\left\|w\right\|}\int_{-\infty }^{\infty }{\left|w\left(t\right)\right|}^{2}{\left(t-t_{*}\right)}^{2}dt\text{,}$$

(43) $${\Delta_{f}}^{2}\equiv \frac{1}{\left\|\overset{ˆ}{w}\right\|}\int_{-\infty }^{\infty }{\left|\overset{ˆ}{w}\left(f\right)\right|}^{2}{\left(f-f_{*}\right)}^{2}df\text{.}$$

The uncertainty principle states that

(44) $$\Delta_{t}\Delta_{f}\geq \frac{1}{4\pi }\text{.}$$

This means that in order to gain good time resolution, a narrow window should be used, but that, on the other hand, good frequency resolution and detection of low frequencies demands wide windows.

#### 4.1.3 The discrete Fourier transform (DFT)

In order to apply the Fourier transform to real signals, we have to use the discrete Fourier transform. Suppose that the original signal has a finite window of length $T=Nt_{s}$ and is sampled at discrete points $jt_{s}$, where j=0,…,N−1. The discrete Fourier transform of the signal

(45) $$G\left(f_{k}\right)=\overset{N-1}{\underset{j=0}{\sum }}g\left(jt_{s}\right)e^{-2\pi ıjk/N}$$

is defined only for discrete frequencies, $f_{k}=k/T$ where k=0,…,N−1. The frequency resolution is determined by the length of the signal as $\Delta_{f}=1/T$ and the upper frequency limit $f_{\max}$ equals $t_{s}/2$.

### 4.2 Wavelet analysis

Wavelet analysis is a scale-independent method in terms of frequency. It uses a mother wavelet which is based on functions of various scales. In the present case, we use the Morlet mother wavelet because of the ease with which scale can be converted to frequency. Within the uncertainty principle, it gives optimal time resolution for high frequencies, and optimal frequency resolution among the low frequency components. It can be written as

(46) $$\psi \left(u\right)=\frac{1}{\sqrt{\pi }}e^{-iu}e^{-u^{2}/2}\text{.}$$

By use of a scaling factor s and a centred time t, a family of nonorthogonal basis functions is obtained as

(47) $$\Psi_{s,t}\left(u\right)={\left|s\right|}^{-1/2}\psi \left(\frac{u-t}{s}\right)\text{.}$$

The continuous wavelet transform of a signal g(u) is then defined as

(48) $$\overset{˜}{g}\left(s,t\right)=\int_{-\infty }^{\infty }{\overset{¯}{\Psi }}_{s,t}\left(u\right)g\left(u\right)du\text{,}$$

where $\overset{¯}{\Psi }$ represents the complex conjugate of Ψ. Thus any specific scale is avoided and the analysis becomes scale independent in terms of frequency. The spectral function $\overset{˜}{g}\left(s,t\right)$ is complex and can be expressed in terms of its amplitude and phase as $\overset{˜}{g}\left(s,t\right)=r\left(s,t\right)\exp\left(ı\theta \left(s,t\right)\right)$. The phase θ(s,t) is considered as an instantaneous phase of the oscillation of frequency scale s at time t[113].

The energy density of the signal in the time scale domain is expressed as

(49) $$\rho \left(s,t\right)=C^{-1}s^{-2}{\left|\overset{˜}{g}\left(s,t\right)\right|}^{2}$$

according to Kaiser [133]. The total energy of the signal g(u) is

(50) $$E_{tot}=\left\|g\right\|=C^{-1}\int \int \frac{1}{s^{2}}{\left|\overset{˜}{g}\left(s,t\right)\right|}^{2}dsdt\text{.}$$

Then energy in a frequency interval from $f_{i2}$ to $f_{i1}$, as introduced in Section 4.3.1, is expressed as

(51) $$E_{i}\left(f_{i1},f_{i2}\right)=\frac{1}{\left(f_{i2}-f_{i1}\right)\left(t_{2}-t_{1}\right)}\int_{1/f_{i1}}^{1/f_{i2}}\int_{t_{1}}^{t_{2}}\frac{1}{s^{2}}{\left|\overset{˜}{g}\left(s,t\right)\right|}^{2}dsdt\text{.}$$

If we use the relationship s=1/f and $ds=-df/f^{2}$, we can easily derive the following equation:

(52) $$E_{i}\left(f_{i1},f_{i2}\right)=\frac{1}{\left(t_{2}-t_{1}\right)}\int_{f_{i2}}^{f_{i1}}\int_{t_{1}}^{t_{2}}{\left|\overset{˜}{g}\left(f,t\right)\right|}^{2}dfdt={\left\|\overset{˜}{g}\right\|}^{2}\text{.}$$

We can recover $\left\|g\right\|={\left\|\overset{˜}{g}\right\|}^{2}$ in analogy to Plancherel’s theorem.

The time- and frequency-averaged amplitude, or wavelet amplitude, in a frequency interval from $f_{i2}$ to $f_{i1}$ is expressed as

(53) $$A_{i}\left(f_{i1},f_{i2}\right)=\frac{1}{\left(f_{i2}-f_{i1}\right)\left(t_{2}-t_{1}\right)}\int_{fi2}^{f_{i1}}\int_{t_{1}}^{t_{2}}\left|\overset{˜}{g}\left(f,t\right)\right|dfdt\text{.}$$

If we use the relationship s=1/f and $ds=-df/f^{2}$, we quickly arrive at the following equation:

(54) $$A_{i}\left(f_{i1},f_{i2}\right)=\frac{1}{\left(f_{i2}-f_{i1}\right)\left(t_{2}-t_{1}\right)}\int_{1/f_{i1}}^{1/f_{i2}}\int_{t_{1}}^{t_{2}}\frac{1}{s^{2}}\left|\overset{˜}{g}\left(s,t\right)\right|dsdt\text{.}$$

Bračič and Stefanovska denoted the averaged amplitude as the *absolute amplitude* [134].

The relative amplitude and energy are defined as the ratios of each of those quantities within a given frequency interval to those within the total frequency interval, in the following way:

(55) $$a_{i}\left(f_{i1},f_{i2}\right)=\frac{A_{i}\left(f_{i1},f_{i2}\right)}{A_{tot}}\text{,}$$

(56) $$e_{i}\left(f_{i1},f_{i2}\right)=\frac{E_{i}\left(f_{i1},f_{i2}\right)}{E_{tot}}\text{.}$$

#### 4.2.1 Frequency resolution

Suppose that the mother wavelet has its centre of gravity at $t_{0}$, $f_{0}$, in time and frequency and that the corresponding deviation is $\Delta_{t_{0}}$ and $\Delta_{f_{0}}$. The scaled mother wavelet $\Psi_{s,t}$ has its centre at $st_{0}$ and a deviation $s\Delta_{t_{0}}$ according to Eq. (47). The centre of $\Psi_{s,t}$ in the frequency domain is expressed as

(57) $$f\left(s\right)=\frac{1}{s}f_{0}\left(s\right)\text{,}$$

and the corresponding standard deviation as

(58) $$\Delta_{f\left(s\right)}=\frac{1}{s}\Delta_{f_{0}\left(s\right)}\text{.}$$

Then the local information around f is given in the frequency interval

(59) $$\left[f_{0}/s-\Delta_{f_{0}\left(s\right)}/2s,f_{0}/s+\Delta_{f_{0}\left(s\right)}/2s\right]\text{.}$$

**Fig. 14:**
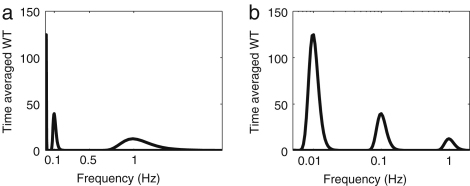
Time-averaged wavelet amplitude for simple sine waves (see the text) plotted (a) on a linear scale and (b) on a semi-log scale.

The ratio between the centre frequency f(s) and bandwidth $\Delta_{f\left(s\right)}$

(60) $$\frac{\Delta_{f\left(s\right)}}{f\left(s\right)}=\frac{\Delta_{f_{0}\left(s\right)}}{f_{0}\left(s\right)}$$

is independent of the scaling s. This property can be seen if the time averages of wavelets of simple sine waves, sin(2πt), sin(0.2πt) and sin(0.02πt), are plotted on linear and semi-log scales as shown in Fig. 14. On the semi-log scale, the width of the peak appears to be the same although on the linear scale it looks quite different.

**Fig. 15:**
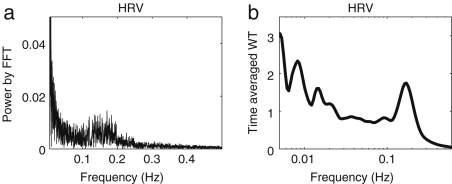
FFT power spectra for simple sine waves (see the text) plotted (a) on a linear scale and (b) as the time-averaged wavelet amplitude plotted on a semi-log scale, from the same HRV signal.

Fig. 15 compares the frequency resolution achieved when an HRV signal is analysed using either the FFT or the wavelet transform. As described in Section 1.4, the frequency bands of HF (0.15–0.40 Hz), LF (0.04–0.15 Hz) and VLF (0.003–0.04 Hz) have been identified mainly by use of the FFT. The wavelet transform enables some additional peaks to be distinguished, which will be discussed in Section 4.3.1.

#### 4.2.2 Energy and amplitude

Let us see what the energy and amplitude of the wavelet are. As described below, our frequency interval of interest is from 0.005 to 2.0 Hz (see Table 1). According to these divisions of frequency intervals, we calculated the energy and amplitude of the sine waves, sin(2πt), sin(0.2πt) and sin(0.02πt), by wavelet analysis. For the three cases, the total energies are the same. This reflects the fact that the total energy equals $\int {\left|g\left(u\right)\right|}^{2}du$. The absolute energy within a certain interval depends on the square of the amplitude of oscillation and does not depend on the frequency. In the case of Bsin(ωt), the total energy of the wavelet depends only on $B^{2}$, but not on ω. Then the relative energy in the ith interval is proportional to ${\left|B_{i}\right|}^{2}/\sum_{j=1}^{6}{\left|B_{j}\right|}^{2}$.

On the other hand, the amplitude of the wavelet is affected not only by the amplitude B but also by the frequency ω. To illustrate this, we use three sine functions whose total amplitudes differ. The higher the frequency is, the higher the total amplitude becomes. However, if we calculate the absolute amplitude in each interval, a higher frequency produces a lower amplitude. In the case of sin(2πt), the total amplitude is 2.7, and $A_{1}$, which is averaged from 0.6 to 2.0 Hz, is 3.9, whereas in the case of sin(0.2πt), the total amplitude is 0.9, and $A_{3}$, which is averaged from 0.052 to 0.145 Hz, is 18. This is because the wavelet has the property that $\Delta_{f}/f$ is constant, as seen in Fig. 14b. If two frequencies, $\omega_{1}$ and $\omega_{2}$, lie in different intervals $i_{1}$ and $i_{2}$, and if the two oscillations have the same amplitude, the wavelet amplitude of the lower frequency $A_{i_{1}}$ is higher than that of the higher frequency $A_{i_{2}}$. If the two frequencies lie in the same interval such that sin(2πt) and sin(2.4πt), the relative amplitude returns the same value in the two cases, which is of course obvious from its definition. But if there are several peaks in different intervals, the interpretation of relative amplitude is much more complicated because the information about amplitude and frequency in the different intervals is combined.

### 4.3 Application to cardiovascular signals

#### 4.3.1 Components that modulate HRV

It is interesting to compare the Fourier transform and evolutive autoregressive (AR) spectral analyses, which are frequently used with HRV signals, with the wavelet transform. For the Fourier transform, the frequency resolution Δf is determined by the window length and is constant for all frequencies. For that reason, it was concluded that Fourier methods are inadequate for the location of peaks in the low frequency interval. In contrast to the Fourier transform case, Δf/f for the wavelet transform is constant. Therefore the relative frequency resolution remains the same over all frequency intervals. The absolute frequency resolution Δf for the wavelet transform is actually much better in the low frequency interval than in the high frequency interval, as shown in Fig. 15. Because of the wide frequency range of the intervals in Table 1, the wavelet transform is more suitable than the Fourier transform.

Autoregressive spectral estimation avoids the problem of frequency discretization. In this method, a model of the time series is first built, and the spectrum of the model is considered as an estimate of the spectrum of the original time series. Linear models of different order are used to represent measured signals. An advantage of the wavelet transform compared to AR estimation is that it is calculated directly from data, and does not need modelling. The limitations of linear modelling, and the choice of model order, are thus avoided.

Lotrič et al. [94] discussed the relative resolution at low frequencies provided by the AR, FFT and wavelet methods as applied to HRV signals. They concluded that the wavelet transform yields the best low frequency resolution.

In what follows, we will be interested in the frequency intervals listed in Table 1 together with the physiological processes that are believed to give rise to them. Note that these oscillatory components not only modulate the heart rate, but also manifest directly in blood flow signals measured by LDF. It is the latter with which we will be especially concerned.

**Fig. 16:**
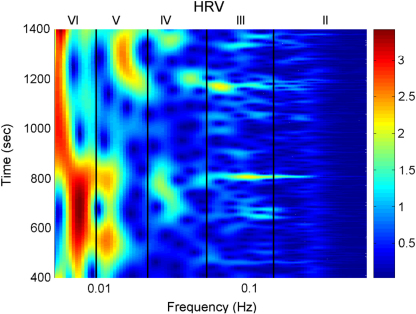
The time–frequency domain of the wavelet transform of HRV.

**Fig. 17:**
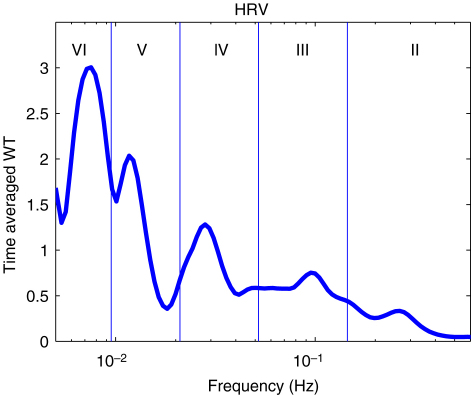
The time-averaged amplitude of HRV.

Blood flow oscillations within frequency intervals I–V were investigated by Stefanovska and co-authors [5,99,134]. The present study also considers a lower frequency interval VI that was identified more recently [101]. The amplitude of the wavelet in the time–frequency domain, and the time-averaged wavelet spectrum are presented in Figs. 16 and 17, respectively. The physiological origins of these spectral peaks have been thoroughly investigated through several different studies [5,78,99–105]. A brief summary of these studies and conclusions drawn from them can be found in a recent status paper by Stefanovska [62]. The existence of the spectral peaks has also been confirmed in a number of independent LDF blood flow studies [135–141]. We now review the intervals briefly. They are:

I Around 1 Hz, corresponding to cardiac activity. The basic frequency near 1 Hz in the ECG signal, which dominates in the blood pressure, corresponds to the heart rate. At rest, it varies from 0.6 in sportsmen to 1.6 Hz in subjects with impaired cardiovascular systems. The effect of the heart pumping is manifested in the vessels.
II Around 0.2 Hz, corresponding to respiratory activity. Following Hales’ discovery of RSA, it has been the subject of many subsequent investigations [142–145]. Modulation in this frequency interval corresponds closely to the respiratory signal as shown in Fig. 2, and the instantaneous respiratory frequency corresponds well to the peak in the frequency domain of HRV wavelet analysis.
III Around 0.1 Hz, corresponding to myogenic activity. The heart and respiratory activity serve as pumps that drive blood through the vessels. The latter are themselves also able to help control blood flow via a mechanism known as myogenic autoregulation. The vascular smooth muscles contract in response to an increase of intravascular pressure, and relax in response to a decrease of pressure [146,147]. Spontaneous activity recorded in microvascular smooth muscle cells was shown to lie in the range 4–10 events per minute (0.07–0.1 Hz) [148]. It was suggested that these waves originate locally from intrinsic myogenic activity of smooth muscle cells in resistance vessels [102,149–155]. Wavelet analysis has shown that the amplitude of myogenic oscillations is increased by exercise [102,156] and decreased by local cooling [113].
IV Around 0.04 Hz, corresponding to neurogenic activity. The autonomous nervous system innervates the heart, lungs and blood vessels, except capillaries. The continuous activity of the autonomous nervous system serves to maintain the basal level of contraction of the vessels. The nerves cause the release of substances that affect the activities of smooth muscles, leading in turn to changes in the vessels’ radii and resistance. Thus the nervous system takes part in vasoconstriction [157]. The peak around 0.03 Hz has been observed in blood pressure, blood flow and HRV signals. It was hypothesized to originate either from metabolic [158] or from neurogenic activity [159]. Kastrup et al. [153] found out that the oscillation around 0.03 Hz disappeared following local and ganglionic nerve blockade in chronically sympathectomized tissue in human. They suggested that this oscillation is a vascular reaction of neurogenic origins. In an LDF study [160] of rabbit skeletal muscle tissues, the oscillations of frequency of 1–3 per minute were suggested as being under neurogenic control. By use of wavelet analysis, it was confirmed that this frequency range is associated with sympathetic nerve activity [104,161]. It was found that there were significantly lower oscillation amplitudes on flaps of transplanted skin, as compared to those for intact skin, in this frequency interval [105]. Bajrović et al. also observed a significant change before and after denervation in rats [162]. An independent study has confirmed these findings by simultaneous measurements of LDF signals on the surfaces of a free latissimus dorsi myocutaneous flap and on the adjacent intact skin of a healthy limb [139]. Recent studies of the effects of local anæsthesia by Landsverk et al. [161] have confirmed the connection between sympathetic activity and the spectral peak in interval IV.
V Around 0.01 Hz, corresponding to NO-related endothelial activity. The blood supplies the cells with nutrients and removes the waste products of their metabolism while circulating around the circuit of vessels. The substances related to metabolism such as O_2_ or CO_2_ have a direct effect on the state of contraction of the vascular musculature. The control of the blood flow based on the concentrations of metabolites is termed metabolic regulation. By simultaneous iontophoretic application (see Appendix Sections B.2.3(b) and B.4.2) of acetylcholine (ACh, an endothelial-dependent vasodilator) and sodium nitroprusside (SNP, endothelial independent), Stefanovska and Kvernmo and co-authors have shown that the oscillations around 0.01 Hz apparently originate from endothelial activity [99,100,103,161,163]. The layer of endothelial cells serves as a barrier between the blood and the tissues of vessels, and controls the contraction and relaxation of smooth muscle by releasing various substances. It seems that metabolic regulation of the blood flow is mediated by the activity of endothelial cells through adjustment of the concentrations of various substances. Nitric oxide (NO) is one of the most important vasoactive substances. It was reported that the interval V was modulated by the inhibition of NO synthesis of the endothelium [100], suggesting that this interval is related to NO from the endothelium. An independent study has confirmed that the oscillations in this interval are NO dependent [136].
VI Around 0.007 Hz, apparently corresponding to NO-independent (probably prostaglandin-dependent) endothelial activity. This interval was not identified in some of the earlier studies, probably because 20 min recordings provided insufficient low frequency resolution and these oscillations were filtered out during data pre-processing. However, a strong peak was later observed around 0.007 Hz [101,113] and is clearly evident in the present work. It was found that the wavelet amplitude at the corresponding frequencies differs between healthy subjects and heart failure patients when ACh is iontophoretically introduced [163].

Note that interval I is not shown in Fig. 17. The HRV signals are determined according to R-peaks as explained in Section 2.3.1. The interval between sequential R-peaks is usually around 1 s. According to Eq. (19), the sampling frequency of the HRV signal is also around 1 Hz. This means that the HRV signals do not have enough sampling points to enable the frequency of interval I to be resolved.

For the calculations in this section, the scaling s is used from 0.5 to 200 with a factor 1.05.

On the basis of the use of the FFT [164], it was reported that the powers of HF (0.15–0.4 Hz) and LF (0.04–0.15 Hz) are significantly lower in elderly subjects than in young subjects. Lotrič et al. [94] studied the effects of ageing on activity by using the wavelet transform within the frequency intervals from I to V in Table 1. In the latter investigation, a decrease with age was observed in all the intervals. There were several differences between the earlier study [94] and our present one: (i) the present measurements allow spectral calculations in intervals II–VI, compared to intervals II–V earlier; (ii) the healthy subject group here is larger; (iii) the larger numbers now allow us to separate the effect of gender; (iv) the subjects in [94] were all Slovenian, whereas here they are mostly British. We note that Vigo et al. [165] reported a decrease of wavelet power with age, albeit using a different definition of intervals within 0.003–0.4 Hz.

**Fig. 18:**
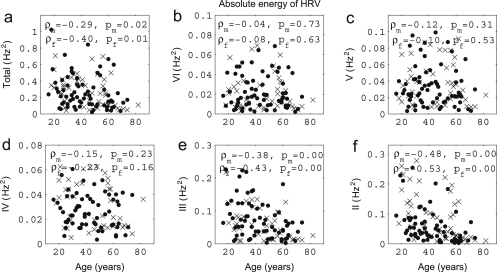
Energy in HRV as a function of age. (a) Total energy. Absolute energy in intervals (b) VI, (c) V, (d) IV, (e) III, and (f) II. The filled circles represent males, and crosses represent females.

**Fig. 19:**
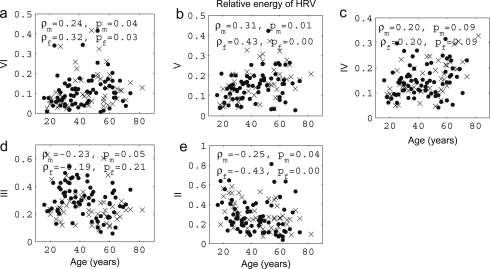
Relative HRV energy in frequency intervals II–VI as functions of age: (a) VI, (b) V, (c) IV, (d) III, (e) II. The filled circles represent males, and the crosses represent females.

We now present new data and their analyses. The effects of ageing on the absolute energy within each interval except I are shown in Fig. 18, and those on relative energy in Fig. 19.

Here, and in what follows, we take p<0.05 as indicating statistical significance (see Appendix C).

It can be seen that total energy decreases significantly with age for both males (ρ=−0.29, p=0.02) and females (ρ=−0.40, p=0.01), corresponding to significant decreases with age in the standard deviation of HRV as shown in Fig. 3. These decreases of total energy come from the significant decreases in absolute energy in intervals II and III both for males and for females. Absolute energy in interval II decreases significantly with age for both males (ρ=−0.48, p=0.00) and females (ρ=−0.53, p=0.00); absolute energy in interval III also decreases significantly with age for both males (ρ=−0.38, p=0.00) and females (ρ=−0.43, p=0.00). Absolute amplitudes in intervals IV, V and VI do not show significant age-related changes.

The relative energy in interval VI and V (endothelial) increases significantly for males (VI: ρ=−0.24, p=0.04 and V: ρ=−0.31, p=0.01) and for females (VI: ρ=−0.32, p=0.03 and V: ρ=−0.43, p=0.00). In interval III (myogenic), it decreases significantly for males (ρ=−0.23, p=0.05). Relative amplitudes decrease in interval II both for males (ρ=−0.25, p=0.04) and for females (ρ=−0.43, p=0.00).

Gender differences were observed in interval II for HRV, and these are summarized in Table 2. In the younger population below 40 years (p=0.01) females are higher than males in terms of absolute energy, but the two groups are the same in the older population above 55 years (p=0.02). In the case of relative amplitude, there are significant differences in interval II in the younger population (p=0.05). This means that RSA is relatively (and absolutely) stronger for females than for males. Physiological reasons for these gender effects are a matter for discussion. But the results indicate that the gender is an important factor to take into account in studies of HRV.

Note that there are some differences between these results and those of [94]. In particular, we see some evidence for increases with age of the relative energy in intervals IV, V, VI with corresponding decreases in intervals II, III–although not all trends are statistically significant. The differences are probably attributable to the differences between the studies themselves (see above). We note that Choi et al. [166] reported that ethnicity can affect the power of HF and LF. These results indicate that characteristics of subjects such as their gender, nationality and age should be carefully considered in conducting measurements and in drawing conclusions from the results.

#### 4.3.2 Oscillatory components in the blood flow signal

In this section, we discuss the oscillatory components in blood flow signals, measured according to the procedure in Appendix B.

**Fig. B.4:**
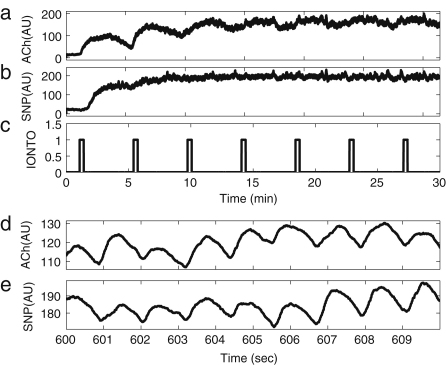
Blood flow in arbitrary units measured with (a) ACh and (b) SNP, over 30 min. (c) The corresponding iontophoretic current pulses. Parts (d) and (e) show 10 s samples of the recordings in (a) and (b) respectively.

As outlined in Appendix A.2, the blood circulates around the closed loop provided by the vascular system. The cardiac output, determined by the product of the heart rate and the stroke volume, amounts to about 5 l/min. The oscillations in blood flow propagate from the heart into the microcirculation. Basal blood flow was recorded on the right wrist and inner right ankle; the iontophoresis chambers for ACh and SNP were positioned a few cm apart on the volar side of the left arm. One of the blood flow signals is shown in Fig. B.4.

It is known that ACh induces vasodilation through enhancement of the activity of endothelial cells, but the exact mechanisms are still not fully understood. The involvement of endothelium in ACh-induced vasodilation is the main difference as compared to SNP-induced vasodilation. It was suggested that impaired ACh-induced vasodilation by comparison with SNP-induced vasodilation could be taken as a demonstration of endothelial dysfunction [167]. See more details about the drugs and iontophoresis in Appendices A.6 and B.

#### 4.3.3 Absolute energy

**Fig. 20:**
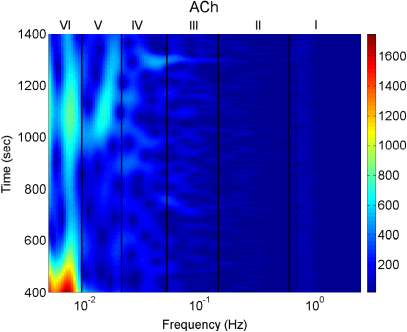
Full time–frequency wavelet transform of the blood flow with ACh.

**Fig. 21:**
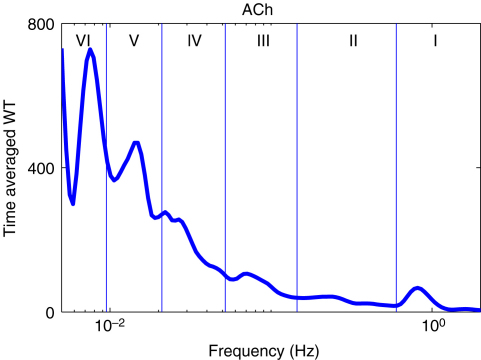
Time-averaged amplitude of the blood flow with ACh.

**Fig. 22:**
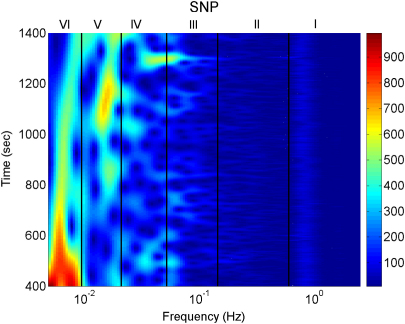
Full time–frequency wavelet transform of the blood flow with SNP.

**Fig. 23:**
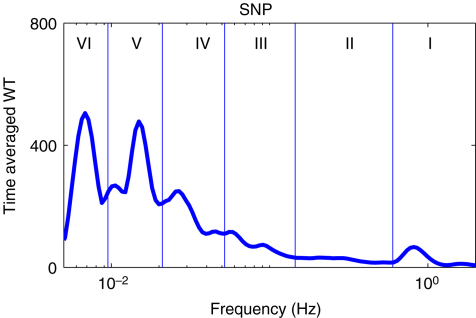
Time-averaged amplitude of the blood flow with SNP.

All of the blood flow signals were resampled from 400 Hz to 10 Hz by averaging 40 points and their lower frequency oscillations below 0.005 Hz were detrended. Then the wavelet analysis was applied to them. The wavelet transform calculated from the signal measured with ACh shown in Fig. B.4a (see Appendix B) is shown in Fig. 20, and its time average is shown in Fig. 21. The wavelet transform calculated from the signal measured with SNP shown in Fig. B.4b (see Appendix B) is shown in Fig. 22, and its time average is shown in Fig. 23. These microvascular blood flow signals stimulated by ACh and SNP are from the same subject. The six peaks, the physiological origins of which have already been discussed in Section 4.3.1, were observed. As we explain in Appendix B, the two vasodilators, ACh and SNP, were applied to assess the change in endothelial function with age. The six peaks still exist in both cases, but their strength differs between endothelial-dependent ACh and endothelial-independent SNP in several intervals, as shown in these wavelet results. For example, in this case, the peak at the lowest frequency for ACh is higher than that for SNP. We again emphasize that we measure the signals with ACh and SNP in close proximity (2–5 cm apart), over similar vasculature, on the same person, simultaneously. Thus the differences come from the different actions of the two substances. As discussed above, their influence on the individual oscillatory components has been investigated in many different studies.

**Fig. 24:**
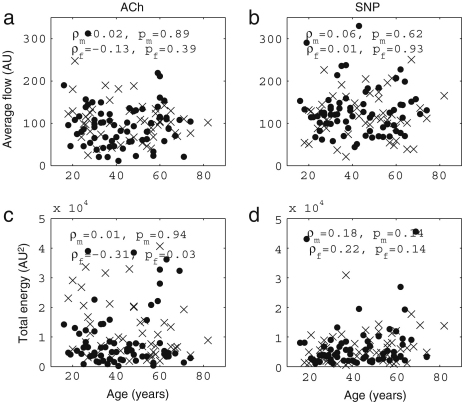
Age-related changes in average flow and total energy. Correlations between age and (a) average flow with ACh, (b) average flow with SNP, (c) total energy with ACh and (d) total energy with SNP. The filled circles represent males, and the crosses represent females.

**Fig. 25:**
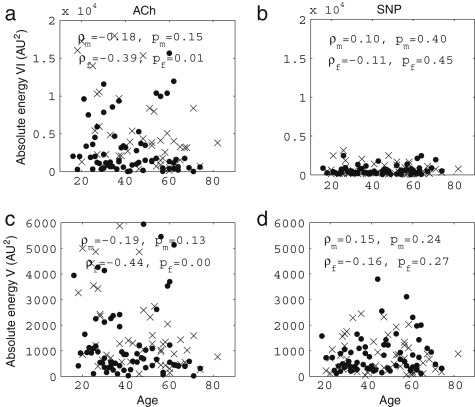
Correlations of absolute energy with age for ACh in intervals (a) VI, (c) V and for SNP in intervals (b) VI, (d) V. The filled circles represent males, and the crosses represent females.

The degree of endothelial activity can be assessed from the wavelet energy in endothelial-associated intervals. Age-related changes in average flow and total energy are shown in Fig. 24, and the absolute energy of endothelial-related intervals in Fig. 25. In fact, average flow does not change with age with either ACh or SNP. The total amplitude with ACh decreases significantly with age for females. It is because the absolute energy in intervals VI and V decreases significantly with age only for females. In fact there are significant gender differences for ACh in intervals VI and V as shown in Table 4. Young females have higher energy in the endothelial-related intervals than young males.

The differences of absolute energy in intervals VI and V between ACh- and SNP-influenced signals are summarized in Table 3. For females, the absolute energy with ACh is higher than that with SNP in interval VI in both the younger and aged populations, and the absolute energy with ACh is higher than that with SNP in interval V in the younger population. For males, the absolute energy with ACh is higher than that with SNP in intervals VI and V in the younger population.

We can conclude that, as they age, humans tend to lose the differences in response to ACh and SNP in intervals V or VI.

#### 4.3.4 Relative energy

When we measured blood flow signals, we tried to choose measurement sites such that the density of vessels would be same for all the subjects. However, it was impossible to get exactly the same density because we could not see the microvasculature under the skin, and because every subject has a different condition of their skin. For this reason, relative energy was calculated to provide a normalized value in each interval by dividing the absolute energy by the total energy. Relative energy has an important meaning related to DFA as explained in Section 4.4.2.

**Fig. 26:**
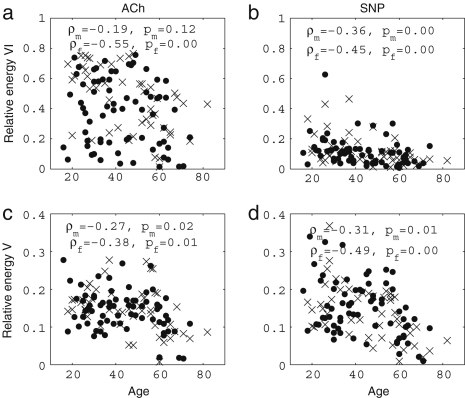
Correlations of relative energy with age for ACh in intervals (a) VI, (c) V and for SNP in intervals (b) VI, (d) V. The filled circles represent males, and the crosses represent females.

The age-related changes in relative energy in interval VI and V with ACh and SNP are shown in Fig. 26. There is a trend that the relative contribution decreases in intervals VI and V. The differences between ACh- and SNP-influenced results for relative energy are summarized in Table 5, and the gender difference in Table 6. With regard to the two substances, the relative contribution of interval VI (endothelial) is higher for ACh than for SNP. With regard to gender differences, females are higher in interval VI in the younger population. However these gender differences disappear in the aged population.

These differences between ACh and SNP may imply that the vessels of females and young males vasodilate more readily at low frequency through endothelial mechanisms rather than through smooth muscles directly. An important result from wavelet analysis is thus that there are the differences related to age and gender in how the vasculature reacts to these vasodilators.

### 4.4 Discussion

The observation that the vessels of females tend to react to ACh more than to SNP is interesting. It may indicate that, for females, the activities of endothelium dominate in causing vasodilation. Are these differences related to the fact that females have fewer cardiovascular problems than males? If so, how?

#### 4.4.1 Age-related changes in oscillatory components

A difference between the spectral energies with ACh and SNP was observed in intervals VI and V, especially for younger females. It is thought that higher energies in these intervals were produced by the endothelial activities and that young females have higher endothelial function than younger males and aged subjects. It is well known that younger females have less cardiovascular risk than males and aged females. Our results support the idea that the higher endothelial activity which generates the oscillations in interval V and VI leads to the healthier cardiovascular function. It may be assumed that the vessels tend to lose their elasticity and ability to dilate spontaneously, through the endothelial response decreasing with age.

The results in the other intervals from I to IV are also important in understanding how ageing affects the blood circulation, but we omit them on account of their complexity. Rather our present aim is to show how we can make use of the wavelet transform, especially for revealing low frequency intervals.

In summary, ageing brings a decrease of endothelial oscillatory activity in blood flow.

#### 4.4.2 The relationship with complexity analysis

It can be seen that HRV signals decrease in amplitude as people get older. The reason lies in the decreases in RSA and myogenic effects with increasing age. The decrease in RSA is well known, and our result is in agreement with that of the previous study [94]. The ways in which the couplings between cardiac and respiratory, and cardiac and myogenic, systems change with age remain unknown, however, and further studies are needed.

**Fig. 27:**
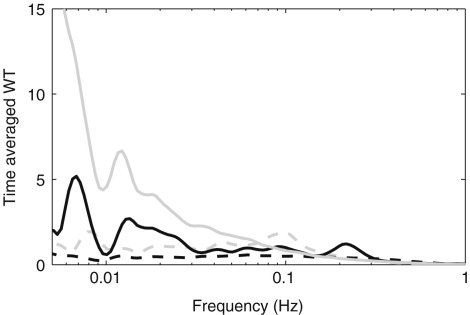
The time-averaged wavelet amplitude of white noise (black, dashed line), Brown noise (grey, full), the HRV of a young female (grey, dashed), and the HRV of an aged female (black, full). The corresponding time series are shown in Fig. 8. The HRV of a young female is closer to white noise than that of an aged female because of stronger fluctuations in the higher frequency intervals.

Now we discuss the relationship between the results of wavelet analysis of HRV, and those of the complexity analysis in the previous section. As we have already discussed, the HRV signals of younger people are more complex than those of aged people. It is to be expected that the signals of young people are closer in shape to white noise, whereas those of aged people are closer to Brown noise in the time scale from 10 to 50 s. To display the differences between the signals, white noise, Brown noise, and the HRV of a young and an aged person are shown in Fig. 27. In each case the wavelet spectrum was calculated from a 100 s segment and time averaged. The result for Brown noise looks smoother than that for white noise because Brown noise has a higher ratio of slow oscillations to fast oscillations than white noise. The HRV of the aged female also looks smoother than that of a young female, for the same reason.

**Fig. 28:**
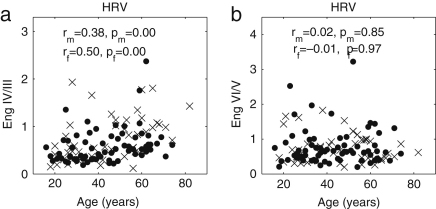
Energy ratios of HRV as functions of age: (a) interval IV over III and (b) VI over V. The filled circles represent males and the crosses represent females.

The range from 10 to 50 s, where the exponent $\alpha_{i}$ increases significantly with age, corresponds to intervals III and IV. It can be assumed that these ageing effects in $\alpha_{i}$ are attributable to the significant increase with age in the ratio of the wavelet energy in the slower oscillations in interval IV to the wavelet energy in the faster oscillations in interval III: for males (ρ=0.38,p=0.00), and for females (ρ=0.50,p=0.00), as shown in Fig. 28a. The age-related changes in $\alpha_{l}$ cannot be seen in the longer time scales from 50 to 300 s, which correspond to interval V and VI. This could be because the ratio of the wavelet energy of the slower oscillations in interval VI to that of the faster oscillations in interval V does not change significantly with age for either males (ρ=0.02,p=0.85) or for females (ρ=−0.01,p=0.97), as shown in Fig. 28b.

## 5 The cardio-respiratory interaction

The cardiac and respiratory systems are known to be coupled in a number of different ways [168]. These include e.g. neurological [169] and mechanical [170] mechanisms. In the previous section, we discussed one consequence of the cardio-respiratory interaction, the modulation of the heart rate by the respiratory system, as well as the modulation by other physiological processes. In this section, we will discuss another result of the interaction between the cardiac and respiratory systems, cardio-respiratory synchronization. The phenomenon has been reported in the study of anæsthetized rats [29], young healthy athletes [27,28], infants [171], healthy adults [32,172–174] and heart transplant patients [32]. As discussed in [173,175], modulation and synchronization are competing processes. In this section, we study the effect of ageing on cardiovascular synchronization and compare it with the results of the other sections.

As we saw in Section 2.1, in the case where oscillators have weak coupling, or there is a weak external force, the perturbation influences only phase. This means that the oscillation can be described by only one variable, the phase. We now discuss phase synchronization under the assumption that the cardio-respiratory interaction is weak enough to be described by phase dynamics.

### 5.1 Theory of a pair of coupled oscillators

In this section, we discuss the case where two almost identical oscillators interact with each other weakly. Their dynamics is given by

(61) $$\frac{dX_{1}}{dt}=F\left(X_{1}\right)+\delta F_{1}\left(X_{1}\right)+V_{12}\left(X_{1},X_{2}\right)\text{,}$$

(62) $$\frac{dX_{2}}{dt}=F\left(X_{2}\right)+\delta F_{2}\left(X_{2}\right)+V_{21}\left(X_{2},X_{1}\right)\text{.}$$

We suppose the dynamics of two oscillators to be closed. Then F(X) is the common structure for the two oscillators and $\delta F\left(X_{i}\right)$ is the deviation from $F\left(X_{i}\right)$. $V_{21}\left(X_{1},X_{2}\right)$ and $V_{21}\left(X_{2},X_{1}\right)$ represent the interacting terms. Using this expression, the phases of oscillators 1 and 2 can be defined in the same way, on the basis of the dynamics of F(X). As shown above in Section 2.1.1, the dynamics of the oscillators is

(63) $$\frac{d\phi_{1}}{dt}=\omega +\left(U^{*},\delta F_{1}\left(\phi_{1}\right)+V_{12}\left(\phi_{1},\phi_{2}\right)\right)\text{,}$$

(64) $$\frac{d\phi_{2}}{dt}=\omega +\left(U^{*},\delta F_{2}\left(\phi_{2}\right)+V_{21}\left(\phi_{2},\phi_{1}\right)\right)\text{.}$$

We introduce new variables as $\phi_{1,2}=\omega t+\psi_{1,2}$ and, by averaging, the equations for ψ are expressed as

(65) $$\frac{d\psi_{1}}{dt}=\delta \omega_{1}+\Gamma_{12}\left(\psi_{1}-\psi_{2}\right)\text{,}$$

(66) $$\frac{d\psi_{2}}{dt}=\delta \omega_{2}+\Gamma_{21}\left(\psi_{2}-\psi_{1}\right)\text{,}$$

where

(67) $$\delta \omega_{1}=\frac{1}{2\pi }\int_{0}^{2\pi }d\theta \left(U^{*}\left(\theta +\psi_{1}\right),\delta F_{1}\left(\theta +\psi_{1}\right)\right)\text{,}$$

(68) $$\Gamma_{12}\left(\psi_{1}-\psi_{2}\right)=\frac{1}{2\pi }\int_{0}^{2\pi }d\theta \left(U^{*}\left(\theta +\psi_{1}\right),V_{12}\left(\theta +\psi_{1},\theta +\psi_{2}\right)\right)\text{.}$$

**Fig. 29:**
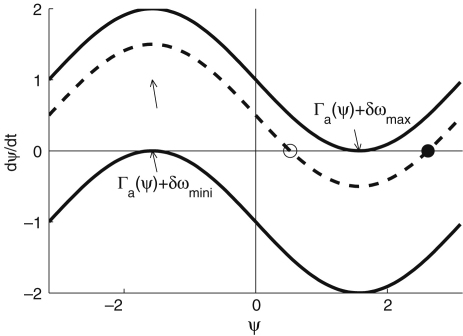
Plot of dψ/dt as a function of ψ. The two solid curves have a minimum or maximum δω which allows the curves to cross dψ/dt=0. The crossing point is the value where phase locking occurs. The dashed curve has δω between the minimum and maximum. The open circle represents a stable fixed point and the filled circle an unstable fixed point.

When the interaction is symmetric, defined by $V_{12}\left(X_{2},X_{1}\right)=V_{21}\left(X_{2},X_{1}\right)=V\left(X_{1},X_{2}\right)$, it is clear that $\Gamma_{12}\left(\psi \right)=\Gamma_{21}\left(\psi \right)=\Gamma \left(\psi \right)$. In that case, the dynamics of the difference of the two phases $\psi =\psi_{1}-\psi_{2}$ is written as

(69) $$\frac{d\psi }{dt}=\delta \omega +\Gamma_{a}\left(\psi \right)\text{,}$$

where $\delta \omega =\delta \omega_{1}-\delta \omega_{2}$ and $\Gamma_{a}\left(\psi \right)=\Gamma \left(\psi \right)-\Gamma \left(-\psi \right)$. Note that $\Gamma_{a}\left(0\right)=\Gamma_{a}\left(\pi \right)=\Gamma_{a}\left(-\pi \right)=0$. If ψ is constant, it means that the two oscillators are synchronized. This synchronization solution ψ=constant corresponds to dψ/dt=0 in Eq. (69). Therefore whether synchronization occurs depends on whether the right hand side of Eq. (69) has a zero solution and whether the zero solution is stable or not. From this, it is concluded that synchronization occurs if δω is within a range shown as Fig. 29. For example, if the coupling function is a simple sine function like Γ(ψ)=−Ksin(ψ), the condition which the frequency difference has to satisfy is |δω/K|<1.

If phase locking occurs and $\psi =\psi_{0}$ (in other words, if $\psi_{0}$ is a stable fixed point), the frequency for both oscillators via entrainment becomes $\omega +\delta \omega_{1}+\Gamma \left(\psi_{0}\right)$, which is equal to $\omega +\delta \omega_{2}+\Gamma \left(-\psi_{0}\right)$.

**Fig. 30:**
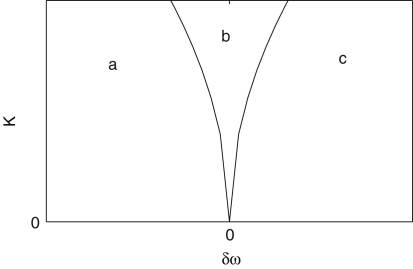
The synchronous region in the K–δω plane. The region b is where phase locking occurs, and it is called the Arnold tongue, whereas in regions a and c phase locking does not occur.

For each coupling strength K, there is a range of δω where phase locking occurs. We can calculate the boundaries of this region in the K–δω plane, which is called the *Arnold tongue* (see Fig. 30).

In the case of n:m synchronization, we can carry through a similar discussion by thinking in terms of $n\psi_{1}$ and $m\psi_{2}$.

If an oscillator is coupled with many oscillators according to

(70) $$\frac{dX_{i}}{dt}=F\left(X_{i}\right)+\delta F_{i}\left(X_{i}\right)+\overset{N}{\underset{j=1}{\sum }}V_{ij}\left(X_{i},X_{j}\right)\text{,}\;\left(i=1,2,\ldots ,N\right)\text{,}$$

the same method as for a pair of coupled oscillators can be applied, and the dynamics of $\psi_{i}\equiv \phi_{i}-\omega t\;\left(i=1,2,\ldots ,N\right)$ is

(71) $$\frac{d\psi_{i}}{dt}=\delta \omega_{i}+\overset{N}{\underset{j=1}{\sum }}\Gamma_{ij}\left(\psi_{i}-\psi_{j}\right)\text{.}$$

In terms of $\phi_{i}$,

(72) $$\frac{d\phi_{i}}{dt}=\omega_{i}+\overset{N}{\underset{j=1}{\sum }}\Gamma_{ij}\left(\phi_{i}-\phi_{j}\right)\text{.}$$

If the equation is of the form

(73) $$\frac{d\phi_{i}}{dt}=\omega_{i}-\frac{K}{N}\overset{N}{\underset{j=1}{\sum }}\sin\left(\phi_{i}-\phi_{j}\right)\text{,}$$

it is known as the Kuramoto model.

### 5.2 Analytical methods

#### 5.2.1 The synchrogram

One way to detect m:n synchronization between respiration and heartbeat is to make a synchrogram. It is constructed by plotting the normalized relative phase of heartbeats within m respiratory cycles according to the following equation:

(74) $$\psi_{m}\left(t_{k}\right)=\frac{1}{2\pi }\phi_{r}\left(t_{k}\right)\;\left(\mathrm{mod}\;2\pi m\right)\text{,}$$

where $t_{k}$ is the time of the kth marked event in the heartbeat and $\phi_{r}\left(t_{k}\right)$ is the instantaneous phase of respiration at the time $t_{k}$. In perfect m:n phase locking, $\psi_{m}$ constructs n horizontal strips in the synchrogram. However, in reality these strips are broadened because of noise. One synchrogram can detect synchronization for only one value of m. For example if we choose m=2, the synchrogram detects only 2:n synchronization. In order to cover all possible synchronization states, we would have to plot synchrograms for all values of m although it would not be practical in reality.

#### 5.2.2 The synchronization index

A synchrogram provides one of the ways to see synchronization visually but it is not adequate for quantifying the synchronization in the presence of noise. It is especially difficult to judge which ratio of synchronization occurs just by inspecting a set of synchrograms with different m. To overcome this weakness, Tass et al. introduced synchronization indices in 1998 [176]. There are two ways to define the synchronization index.

One is based on the conditional probability. We have two phases $\phi_{1}\left(t_{j}\right)$ and $\phi_{2}\left(t_{j}\right)$ defined on the intervals [02πm] and [02πn] respectively, where j is an index of time. Each interval is divided into N bins. We take a particular centred time $t_{c1}$ and decide a certain window length around $t_{c1}$ and call this time interval ‘interval-1’. We take all j such that $t_{j}$ is within the interval-1. Then, for each bin l, 1≤l≤N, we calculate

(75) $$r_{l}\left(t_{c}\right)=\frac{1}{M_{l}}\sum e^{ı\phi_{2}\left(t_{j}\right)/n}$$

for all j, such that $\phi_{1}\left(t_{j}\right)$ belongs to the bin l and $M_{l}$ is the number of points in this bin. If there is a complete n:m dependence between two phases, then $\left|r_{l}\left(t_{c1}\right)\right|=1$, whereas it is zero if there is no dependence. Finally we calculate the average over all bins by application of the following equation:

(76) $$\gamma_{nm}\left(t_{c}\right)=\frac{1}{N}\overset{N}{\underset{l=1}{\sum }}\left|r_{l}\left(t_{c}\right)\right|\text{.}$$

Thus $\gamma_{nm}$ measures the conditional probability for $\phi_{2}$ to have a given value provided that $\phi_{1}$ is in a certain bin at the time $t_{c}$. Then we move the centred time $t_{c}$ to $t_{c}^{\prime }$ and recalculate the index in the same way. In order to find m and n, we need to try different sets of values and select the set that gives the largest index.

The other approach is based on entropy. It is defined by

(77) $$\rho_{nm}=\frac{S_{\max}-S}{S_{\max}}\text{,}$$

where S is the entropy of the distribution of $\Psi_{m,n}=n\phi 1-m\phi 2\;\left(\mathrm{mod}\;2\pi \right)$ and defined as

(78) $$S=-\overset{N}{\underset{k=1}{\sum }}p_{k}\ln p_{k}\text{,}$$

where $p_{k}$ is the probability that $\Psi_{m,n}$ is in the bin k. Note that $S_{\max}=\ln N$, where N is the number of bins. It is normalized in such a way that $0\leq \rho_{nm}\leq 1$, where $\rho_{nm}=0$ corresponds to a uniform distribution (no synchronization) and $\rho_{nm}=1$ corresponds to a Dirac delta-like distribution (perfect synchronization).

### 5.3 Application to cardiovascular signals

#### 5.3.1 The synchronization duration of real data

In this section, we determine the synchronization duration of the data that we measured according to the procedures described in Appendix B. We first introduce a method for evaluating the degree of synchronization in order to be able to discuss the age-related changes in synchronization. We calculate the synchronization index of 1:n and 2:n for cardio-respiratory synchronization for each subject with the window length 5T for 1:n synchronization and 10T for 2:n synchronization where T is the average respiratory period. The reason for this choice of window length is to see the synchronization during the same periods for all subjects, rather than using a fixed time for them all. If the index exceeds 0.95 for a duration longer than 5T for 1:n synchronization, and for 10T for 2:n synchronization, we judge that synchronization occurred during the interval.

**Fig. 31:**
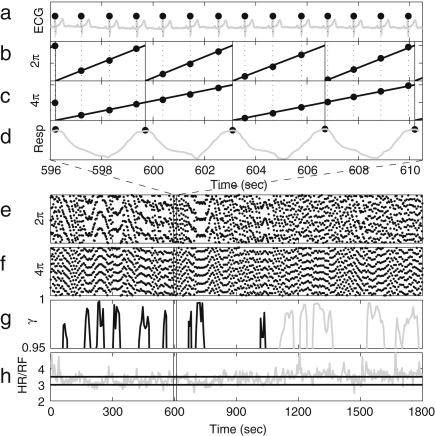
Construction of a cardio-respiratory synchrogram. (a) An ECG signal during a short time segment with its R-peaks marked by small filled circles. (b) The phases (mod 2π) of the respiration signal shown in (d) at the marked R-peaks times in (a) during the time segment. (c) The phases (mod 4π) of the respiration signal shown in (d) at the marked R-peaks times in (a) during the time segment. (d) The respiratory signal during the time segment, with its maxima marked. (e) A synchrogram for 1:n synchronization during the whole measurement period. (f) A synchrogram for 2:n synchronization during the whole measurement period. (g) Synchronization indices γ above 0.95 during the whole measurement period, where the black line represents 1:3 and the grey line represents 2:7 synchronization. (h) The ratio between HRV and RFV during the whole measurement period, where the two lines lie at the ratios 3.0 and 3.5, corresponding to 1:3 and 2:7 synchronization. All are calculated from the same data, as shown in Fig. 2.

When the signal of one subject is exchanged with the signal of another subject, or when a signal is randomized, the synchronization index can occasionally reach high values without real cardiovascular coupling. To be sure that the synchronization comes from a genuine cardiovascular interaction, we set these minimum thresholds for synchronization index and duration. The synchrogram and synchronization index are shown in Fig. 31 together with a demonstration of how to construct a synchrogram. It can be seen that the state of synchronization changes with time and that the synchronization ratio changes spontaneously from 1:3 to 2:7. This kind of synchronization transition is quite common and is seen for all subjects. It is evident that noise disturbs the synchronization and that the synchronization makes frequent transitions from one state to another. Finally we calculate the total duration of synchronization during which the index was beyond 0.95. The synchronization duration is proportional to the ratio of synchronization time to the whole measurement time of 1800 s, which was fixed for all subjects. We therefore consider the synchronization duration as equivalent to the percentage of respiratory periods which are in the state of synchronization during the whole measurement period. With this interpretation, differences in the average respiratory period do not affect the result.

**Fig. 32:**
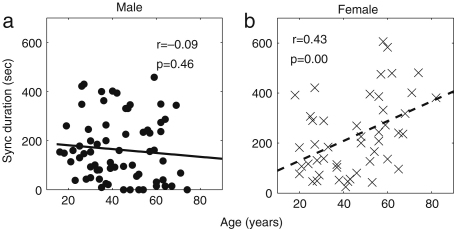
The total synchronization duration of the original data plotted as a function of age for (a) males and (b) females.

The results are shown in Fig. 32. The synchronization duration increases significantly with age for females (ρ=0.42,p=0.00), whereas it does not have any correlation with age for males (ρ=−0.14,p=0.25). Females have a longer synchronization duration than males in the elderly population above 55 years (p=0.00), whereas there is no significant gender difference in the younger population (p=0.86). These results indicate that beyond the age of 55 there is a large, significant increase in the mean extent of synchronization compared to the males at all ages and the younger female group.

#### 5.3.2 The correlation of synchronization duration with HRV and RFV

**Fig. 33:**
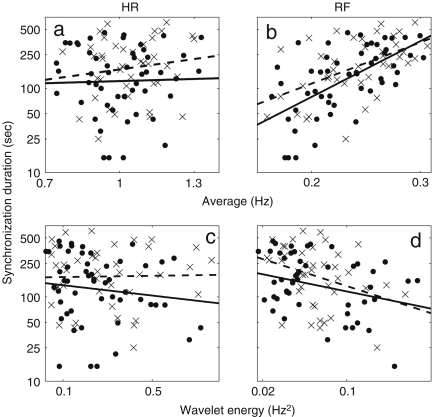
Correlations between the logarithm of the synchronization duration and (a) the average heart rate, (b) the average respiratory rate, (c) the total wavelet energy of heart rate, and (d) the total wavelet energy of the respiratory rate. The filled circles represent males and the crosses represent females.

We first examine possible correlation between the synchronization duration and the heart and respiratory rates. The results are shown in Fig. 33. The logarithm of the synchronization duration has a significant positive correlation with the average respiratory rate, for both males (ρ=0.61,p=0.00) and females (ρ=0.54,p=0.00), and a significant negative correlation with total wavelet energy in RFV for both males (ρ=−0.41,p=0.00) and females (ρ=−0.34,p=0.03). There are no gender-specific differences between the extents of the correlations of these variables. In contrast to the respiratory frequency, the duration of synchronization is independent of the average HR or HRV in both males and females. This reinforces the inference that the duration of synchronization is strongly linked to the respiratory oscillator.

The correlation with average respiratory frequency and synchronization duration could be spurious, i.e. attributable to the algorithm. Bias could arise because the synchronization threshold is proportional to the average respiratory frequency and it is less likely for the synchronization to be maintained over the longer duration. Moreover, it is difficult to compare synchronization times for subjects who have significantly different respiratory frequencies. If we choose a fixed threshold of e.g. 30 s, the average respiratory frequency will not affect the results, but it would be doubtful that we could identify the 2:n synchronization reliably for a person whose average respiratory period is longer than 7.5 s. We need a longer time to judge the synchronization state for the subjects who have longer average respiratory frequencies to compare their state of synchronization. On the other hand, it may be more difficult for a living system to maintain its stationary state for a longer time. However, these results do at least show that a larger standard deviation of respiration (for both genders) leads to increased synchronization duration in the resting state for healthy subjects.

The observation that a bigger standard deviation leads to shorter synchronization epochs corresponds well to our picture that, if the frequency fluctuates dramatically, the parameters easily move outside the Arnold tongue, thus destroying the synchronization. The point has also been discussed in the recent study by Kenwright et al. [174].

Although we could not see any significant correlation between age and duration of synchronization for males, the duration has a significant correlation with the wavelet total energy of respiration, which is *not* significantly correlated with age. The total energy of the heart rate, which decreases significantly with age, does not have a significant correlation with the synchronization duration. For females, it is certain that respiration has a significant effect in causing the synchronization. There is a trend for the average RF to increase with age for females although the correlation is not statistically significant (p=0.09). This means that increases in average respiratory rate are matched by corresponding increases in the variability of that RF. There is no correlation between these variables in the male population. Thus the slight trend for increased RF with age in the female population, along with stronger relationships between RF and RFV in the female population, together with the strong effects of RF and RFV on the duration of synchronization, manifest in a highly significant lengthening in the epochs of synchronization in older females.

#### 5.3.3 Surrogate data

The first point to be settled is whether the synchronization reflects the true cardiovascular interaction, or whether it is just noise, i.e. a random fluctuational phenomenon. This problem was investigated by Toledo et al. [32] by using surrogate data. In the present study, we created surrogate data from the original signals and used them to calculate the duration of “synchronization”. Surrogate data are artificially generated data that mimic some of the statistical properties of the data under study, but not the particular property that is being tested.

Surrogate data use was methodologically introduced into time series analysis as a method of testing for nonlinearity [177]. The basic idea is to compute a nonlinear statistic for the data under study and to do the same for an ensemble of realizations of surrogates that mimic the linear properties of the data studied. If the computed statistics for the original data is significantly different from that obtained from surrogates, one can infer that the data set was not generated by a linear process. Otherwise, the null hypothesis that a linear model fully explains the data must be accepted.

There are several ways of producing surrogate data to meet the needs of studies. For bivariate data, four types were proposed by Paluš [178]:

• IID1 surrogates are realizations of mutually independent IID (independent identically distributed) stochastic processes (white noise) with the same mean, variance and histogram as the series under study. The IID surrogates are constructed by scrambling the original signal, i.e. the elements of the original series are randomly permuted in temporal order and different random permutations are used for the two components of the bivariate series.
• IID2 surrogates are realizations of IID stochastic processes (white noises), which take account of possible cross-dependences between the two components of the bivariate series. In each realization, the same random permutation is used for both components of the bivariate series. The IID surrogates present the null hypothesis of mutually dependent white noise, i.e. the two series are synchronized in a sense of mutual dependence given, e.g., by cross-correlations; but the specific phenomenon as well as other temporal structures are absent.
• FT1 surrogates are independently generated for each of the two components in the bivariate data as realizations of linear stochastic processes with the same power spectra as those under study. The FT1 surrogates are obtained by computing the Fourier transform (FT) of the series; it is then returned to the time domain with unchanged magnitude but with the phases randomized. The FT1 surrogates realize the null hypothesis of two linear stochastic processes which asynchronously oscillate with the same frequencies as the original series under study.
• FT2 surrogates are realizations of a bivariate linear stochastic process that mimics individual spectra of the two components of the original bivariate series as well as their cross-spectrum. When constructing the FT2 surrogates, not only the spectra but also the differences between phases of the Fourier coefficients of the two series for particular frequency bins must be kept unchanged. In this case, the same random number must be added to the phases of both coefficients of corresponding frequency bins. The FT2 surrogates preserve some of the synchronization, if present in the original series, which can be explained by a bivariate linear stochastic process.

In our study, we derived IID1 surrogates from the original cardiac and respiratory signals. The phases of the original signals were determined by the time of marked events according to Eq. (15). The periods between marked events were permuted randomly. For example, if the time of marked events of an original signal is [1,2.5,3.7,5], the periods of original signals are [1.5,1.2,1.3]. Then these periods are randomly permuted like e.g. [1.2,1.3,1.5] and the time of marker events of surrogate is then [1,2.2,3.5,5] and the phases are calculated according to Eq. (15). Different ways of achieving randomization were used for the cardiac and respiratory signals. Finally, the index and duration of synchronization for surrogates were calculated using the same algorithm as for the original data.

**Fig. 34:**
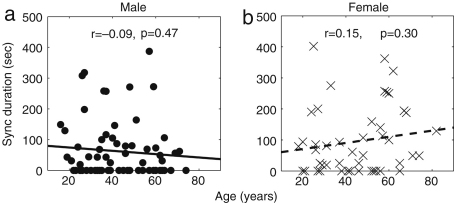
The correlation between the total synchronization duration and age for the surrogate data for (a) males and (b) females.

The results are shown in Fig. 34. Surrogate data still have the same apparent synchronization durations even after being randomized. However, the duration does not increase significantly with age (males p=0.51; females p=0.20) and the correlation with age is lower than the results from the original time series for both genders. This implies that the results from the original data do not arise just on account of noise.

We compared the epochs of synchronization of the original time series and the apparent epochs of synchronization in the surrogate time series for each gender. The original time series have significantly longer synchronization epochs than the surrogate time series (males p=0.00; females p=0.00). This means that the synchronization obtained is real, and not just a coincidence.

### 5.4 The directionality of cardio-respiratory coupling

#### 5.4.1 Methods

The detection of coupling direction has been treated by making use of the amplitudes of the observables and evaluating their mutual predictability [179,180] or mutual nearest neighbours in reconstructed state spaces [181,182], or by information-theoretic approaches [37,183,184]. In order to determine the predominant direction of any coupling between the heart rate and the respiratory rate of the data which we measured, we based our analysis on the permutation information approach described by Bahraminasab et al. [184] and by Paluš and Stefanovska [37].

We first define and discuss briefly the permutation entropy (PE) of a time series. We take $X_{1}\left(t_{i+1}\right),\ldots ,X_{1}\left(t_{i+n}\right)$, and then sort n points into an increasing order. If $X\left(t_{j_{1}}\right)>X\left(t_{j_{2}}\right)>\cdots >X\left(t_{j_{n}}\right)$, $j_{1}j_{2}\ldots j_{n}$ is the ordered type which we get. The possible $j_{1}j_{2}\ldots j_{n}$ is one of n! permutations ($j_{1},j_{2},\ldots ,j_{n}\in 1,\ldots ,n$). We map each n consecutive data points of the time series starting from a different time index i to one of the ordered types out of the n! permutations. We represent all the possible ordered types by $\pi_{1},\pi_{2},\ldots ,\pi_{n!}$. The PE of the time series is then defined as the Shannon entropy

(79) $$H\left(X\right)=-\overset{n!}{\underset{x=1}{\sum }}p\left(\pi_{x}\right)\ln\left(p\left(\pi_{x}\right)\right)\text{,}$$

where $p\left(\pi_{x}\right)$ is the probability distribution of $\pi_{x}$. The PE shows the same behaviour for different values of n. Next, we introduce the directionality of coupling using information-theoretic tools. Consider two time series $X_{1}\left(t\right)$ and $X_{2}\left(t\right)$ representing the observations from two possibly coupled systems. The average amount of information contained in the variables $X_{2}$ concerning the process $X_{1}$ in its future τ time units ahead is quantified by the mutual information $I\left(X_{2},X_{1\tau }\right)=H\left(X_{2}\right)+H\left(X_{1\tau }\right)-H\left(X_{2},X_{1\tau }\right)$, where the entropy H is calculated in the PE sense. $H\left(X_{2},X_{1\tau }\right)$ is called the joint entropy and is expressed as

(80) $$H\left(X_{2},X_{1\tau }\right)=-\overset{n!}{\underset{x_{2}=1}{\sum }}\overset{n!}{\underset{x_{1\tau }=1}{\sum }}p\left(\pi_{x_{2}},\pi_{x_{1\tau }}\right)\ln\left(p\left(\pi_{x_{2}},\pi_{x_{1\tau }}\right)\right)\text{.}$$

If $I\left(X_{2},X_{1}\right)>0$, the processes $X_{1}$ and $X_{2}$ are not independent. In order to infer the directionality of coupling between the processes $X_{1}$ and $X_{2}$, such as the driving influence from $X_{2}$ and $X_{1}$, we need to estimate the net information concerning the τ future time of the process $X_{1}$ contained within the process $X_{2}$. We increment vectors of n points of $X_{1}$ as $X_{1}\left(t_{i}\right)-X_{1}\left(t_{i+\tau }\right),X_{1}\left(t_{i+1}\right)-X_{1}\left(t_{i+1+\tau }\right),\ldots ,X_{1}\left(t_{i+n}\right)-X_{1}\left(t_{i+n+\tau }\right)$, write these vectors $\Delta X_{1}$, and map them to ordered types $\pi_{\Delta x_{1}}$ out of n! permutations for every possible time index i. How much system 2 drives system 1 is measured by the conditional mutual information $I_{21}=I\left(X_{2},\Delta X_{1}|X_{1}\right)$ of the variables $X_{2}$ and $\Delta X_{1}$ given the variable $X_{1}$ which is expressed as $I_{21}=I\left(X_{2},\Delta X_{1}|X_{1}\right)=H\left(X_{2}|X_{1}\right)+H\left(\Delta X_{1}|X_{1}\right)-H\left(X_{2},\Delta X_{1}|X_{1}\right)$, where the conditional entropy $H\left(X_{2}|X_{1}\right)$ is expressed as

(81) $$H\left(X_{2}|X_{1}\right)=-\overset{n!}{\underset{x_{2}=1}{\sum }}\overset{n!}{\underset{x_{1}=1}{\sum }}p\left(\pi_{x_{2}},\pi_{x_{1}}\right)\ln\left(p\left(\pi_{x_{2}}|\pi_{x_{1}}\right)\right)\text{,}$$

and

(82) $$H\left(X_{2},\Delta X_{1}|X_{1}\right)=-\overset{n!}{\underset{x_{1}=1}{\sum }}\overset{n!}{\underset{\Delta x_{1}=1}{\sum }}\overset{n!}{\underset{x_{2}=1}{\sum }}p\left(\pi_{x_{1}},\pi_{\Delta x_{1}},\pi_{x_{2}}\right)\ln\left(p\left(\pi_{x_{2}},\pi_{\Delta x_{1}}|\pi_{x_{1}}\right)\right)\text{.}$$

If $I_{21}=0$, there is no information in $X_{2}$ about the τ future time of the process $X_{1}$, but if $I_{21}>0$, there is some information in $X_{2}$. In the same way, $I_{21}=I\left(X_{1},\Delta X_{2}|X_{2}\right)$ is calculated. Directionality of coupling from 1 to 2 can be written as

(83) $$D_{12}=\frac{I_{12}-I_{21}}{I_{12}+I_{21}}\text{.}$$

There is also another way to calculate the conditional mutual information $I_{21}$ according to Paluš and Stefanovska [37]. We define $\Delta_{\tau }X_{1,2}$ as

(84) $$\Delta_{\tau }X_{1,2}=X_{1,2}\left(t+\tau \right)-X_{1,2}\left(t\right)\text{.}$$

The conditional mutual information is written in the same way as above, $I_{21}=I\left(X_{2},\Delta_{\tau }X_{1}|X_{1}\right)=H\left(X_{2}|X_{1}\right)+H\left(\Delta_{\tau }X_{1}|X_{1}\right)-H\left(X_{2},\Delta_{\tau }X_{1}|X_{1}\right)$, which is obtained by a simple box counting algorithm based on equiprobable marginal bins (marginal equiquantization [185]).

In our study, $X_{1}\left(t\right)$ represents the phase of the heart and $X_{2}\left(t\right)$ represents the phase of the respiration which was reconstructed with a sampling frequency of 10 Hz. We took τ=5,6,…,50 for both PE and equiquantal methods, which means the delay was from 0.5 to 5 s. For each τ, we express mutual information as $I_{12}\left(\tau \right)$. After getting 46 values of $I_{12}\left(\tau \right)$ and $I_{21}\left(\tau \right)$ under the parameter of n=4 for the PE method and 8 bins for the equiquantal method, we took the averages over the values and obtained the final values of $I_{12}$ and $I_{21}$ for each subject for each method as

(85) $$i\left(X_{2}\to X_{1}\right)=\frac{1}{N}\overset{\tau_{max}}{\underset{\tau =\tau_{min}}{\sum }}I_{12}\left(\tau \right)\text{,}$$

where N=46, $\tau_{min}=5$ and $\tau_{max}=50$ in our case. We refer to this i as the intensity of influence. For the results presented below in the next section, we write the intensity calculated by the PE method as $i_{\mathrm{Perm}}$ and intensity calculated by the equiquantal method as $i_{\mathrm{Eqq}}$.

100 sets of randomized surrogate data were analysed in the same way by each method, and the average of 100 surrogate data sets was taken for each subject.

#### 5.4.2 Results

The evidence discussed above shows that the RF exerts a strong influence on the extent of synchronization. This would suggest that the respiratory system is the driving force in the coupling between these two oscillators. Confirmation of this inference was achieved using the permutation information approach to obtain information about the directionality of the coupling.

**Fig. 35:**
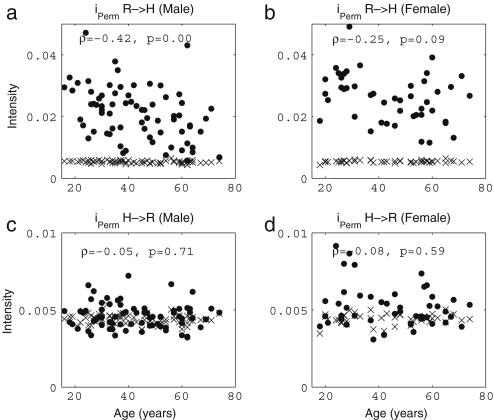
Changes of intensity of the inter-oscillator influence with age calculated by the permutation entropy (PE) method: (a) from respiration to heart for males; (b) from respiration to heart for females; (c) from heart to respiration for males; (d) from heart to respiration for females. Circles are for real data with correlation coefficients ρ and p, and crosses represent surrogate data.

**Fig. 36:**
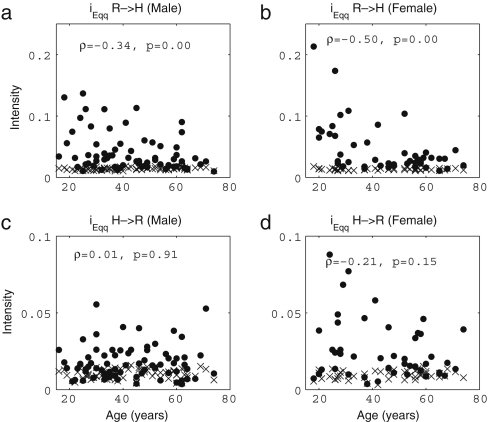
Changes of intensity of the inter-oscillator influence with age calculated by the equiquantal method (a) from respiration to heart for males; (b) from respiration to heart for females; (c) from heart to respiration for males; (d) from heart to respiration for females. Circles are for real data with correlation coefficients ρ and p, and crosses represent surrogate data.

The intensity of influence is much stronger from respiration to heart than in the opposite direction, for both genders, and from both methods, as shown in Figs. 35 and 36.

An age-related decline in the effect of respiration on cardiac activity for males is found by both methods (Figs. 35a and 36a), whereas there are no age-related changes in the effect of cardiac activity on respiration (Figs. 35c and 36c). For females, there is a trend towards a decline with age for the intensities in both directions, as found by both methods, but there is a slight difference between the two methods in the calculated significance (cf. Figs. 35b and 36b, and cf. Figs. 35d and 36d). The p-value of the effect of respiration on cardiac activity found by the equiquantal method is close to the borderline of significance (Fig. 36b). It could therefore be claimed that there is a decline with age for females as well.

The intensity of influence in both directions is significantly larger for females, as compared to males, when calculated by the PE method (p<0.01 from respiration to heart and p=0.01 from heart to respiration), whereas these gender differences were not found to be statistically significant by the equiquantal method.

The decline with age of the intensity of influence from respiration to heart calculated by the PE method is much more pronounced in males than in females (Fig. 35a and b). Thus in females the respiratory system has much more influence on the cardiac system, compared to the case for males, and the effects of ageing compound this difference as the i values in females decline at a slower rate compared to those for the male population. Since this effect was not observed by the equiquantal method, it requires confirmation (or refutation) through further, more detailed, studies of the numerical properties of the two methods.

This possible strong influence of the respiratory system, linked with the trends in respiratory rate and respiratory rate variability in the female population, leads to significant age-related increases in the extent of synchronization between these two oscillators in the female population. There is no age-related change in the extent of synchronization in the male population.

Since these time series are non-stationary, it is to be expected that the intensities of influence will fluctuate with time. We also calculated the time-dependent intensity by dividing each signal into ten 3 min windows, which was the minimum length required to obtain reliable results. Then we counted the length of time when the intensity of influence from the real data was larger than that from the surrogate data for each subject, and plotted these time lengths as a function of age. The results were quite similar to those for the intensity calculated from whole signals (Figs. 35 and 36).

### 5.5 Discussion

There can be no doubt that the cardiac and respiratory systems behave as two oscillators forming a coupled system. Their coupling arises through both mechanical interactions and also through coordinated neurological control mechanisms as part of the overall homeostasis of the whole organism. The physical manifestations of this coupling have been extensively studied in the context of frequency modulation (respiratory sinus arrhythmia—RSA) between the two systems and, more recently, synchronization (frequency and phase coordination) [26–28,32,63,115,142–145,171,173,186]. The evolution of new methodologies in nonlinear dynamics has facilitated the detection of phase synchronization between the two biological oscillators, and these methods have been used to study synchronization in the examples already mentioned above—in athletes, in adult and infant sleep patterns and in anæsthesia [27,28,63,171,186].

We noted that the synchronization ratio is not constant but changes with time. This is because the ratio of HR and RF fluctuates with time as shown in Fig. 31h. It seems to be non-stationary, like the heart rate and respiratory rate. The synchronization ratio is not restricted to 1:n but may also be 2:n. There is the possibility that the ratio is m:n where m is more than 3. However, it is usually difficult to detect synchronization with higher m because it requires a longer window for calculation and the noise component become more significant.

We now discuss these issues in more detail.

#### 5.5.1 Is cardio-respiratory synchronization dependent on the respiratory rate and its variability?

We have used nonlinear methods to assess the relative importance of the two oscillatory systems in allowing synchronization to be achieved. Our data demonstrate that the respiratory rate and variations are strongly related to the duration of synchronization that is observed over the 30 min measurement period. Further analysis of the directionality of coupling between the cardiac and respiratory oscillators also demonstrates that the respiratory system is the major influence with no detectable effect of the cardiac system. Our studies on synchronization link to studies of another interaction between the two systems, namely RSA—the modulation of the heart rate by the frequency of respiration. Models of RSA, including the idea of a ‘respiratory gate’, point to the importance of the respiratory rate in the modulation of cardiac rhythms [187]. In our case, looking at synchronization and coupling between the systems, the respiratory rate is again identified as important. Added to this we have now demonstrated that RF and the extent of the variability in RF are also important factors.

#### 5.5.2 Gender-specific differences in age-related changes in cardio-respiratory synchronization

There are strong gender-specific differences in the coupling of the respiratory and cardiac oscillators. The duration of synchronization is independent of age in males, but the extent of synchronization is significantly higher in older females compared to younger females or males of any age. This effect is not associated with noise, as assessment of the surrogate data set does not result in any association between these two variables. Thus in healthy resting females the extent of synchronization between the cardiac system and the respiratory system increases as they get older and is particularly evident in those over 55 years old.

#### 5.5.3 Gender-based differences in the ageing process

Gender-specific effects of ageing on the human body have been studied at many levels, ranging from the assessment of the length of telomeres at the end of chromosomes through to whole system studies as described here. One interesting feature arising from the telomere studies is that for any ‘biological age’ the telomere lengths are shorter in males compared to females, suggesting that the ageing process advances faster in the male population [188]. Demographic data (with the life expectancy of females being greater than that of males) and studies of other ageing processes including cardiovascular, renal and respiratory systems show the same phenomena [189]. We find that the extent of synchronization in males is independent of age across the whole age-span so the changes observed in the female population are not merely a manifestation of a general delay in the ageing process compared to the case for males. Many differences between the ageing processes in males and females are linked to the beneficial effects of estrogens. In post-menopausal women these benefits are lost, such that eventually the ageing process in females in their ninth and tenth decades would catch up with the ageing process in their male counterparts. On this basis we would expect that the synchronization patterns for females would tend to converge to the male data. In fact the opposite is true; the gender differences between the durations of synchronization are enhanced in later years, not reduced.

#### 5.5.4 Are the gender-based differences linked to changes in the HR or HRV?

What are the origins of this increase in synchronization? In terms of the individual oscillators, the variabilities in the heart rate and the respiratory frequency are independent of age in both males and females (as shown in Fig. 3). In agreement with previously published data, the spectral energy (a measure which is proportional to the standard deviation) of the HRV is significantly negatively correlated with age [7,39,120,190]. This effect can be visualized by consideration of Fig. 18; although the actual average HR does not change, the extent of variability on either side of the line denoting the average value is decreased. This manifestation of the ageing process is probably linked to a decrease in the plasticity of the heart with ageing. This decline is present in both the male and female populations and the extent of this decline does not differ significantly between the male and female subjects. This suggests that the increase in the extent of synchronization in females is not associated with alterations in HRV. Previous studies also revealed that the standard deviation of the heart rate is negatively correlated with the time-averaged synchronization index [6] but the gender of the subjects used in this latter study was not identified.

#### 5.5.5 Are the gender-based differences linked to changes in RF or RFV?

In contrast to the results for HR and HRV in females, the origin of the alterations in the extent of synchronization are to be found in the overriding relationship between the duration of synchronization, RF and RFV. There were no significant relationships between ageing and RF or RFV in either males or females, but within the female population there was a trend towards increased RFV with age. Also in females the average RF is significantly correlated with its own variability. Another factor in the extent of synchronization observed between these two oscillatory systems is the directionality of the coupling between them. In both males and females the directionality is from the respiratory system to the cardiac system and this direction does not change with age. What does change however is the extent of the influence (intensity) that respiration has on HR. As subjects become older, the influence declines, but the starting level is higher in females and the rate of decline is much lower (as gauged by the PE method). Thus in females RF has more influence on the HR than in males. Taken together, all of these effects result in stronger synchronization in the cardio-respiratory systems of older females.

#### 5.5.6 Synchronization in other physiological states

Alternative explanations for the increased duration of synchronization come from other studies looking at duration of synchronization in different physiological settings. In anæsthetized rats, longer synchronization duration was observed (with the concomitant decrease in the standard deviation of respiratory frequency) [29]. Similarly, in non-REM sleep the extent of synchronization was increased whilst in REM sleep the extent of synchronization was decreased compared to that for wakefulness [186]. Longer synchronization times have also been linked to the better physiological condition of athletes as compared to non-athletes [27]. However, it has been shown that synchronization is reduced, or almost destroyed, during the exercise itself [174]. In general, the greater the modulation of HRV, especially by the low frequency components, the lesser the degree of synchronization that occurs. On the basis of the anæsthesia and sleep studies it appears that decreased input from neurological control mechanisms leads to enhanced synchronization. In terms of the directionality of this coupling, our observation of significant coupling from respiration to cardiac activity is in agreement with several earlier studies [36,37,174,184]. In babies the coupling is symmetrical just after birth but, within six months, the influence from respiration to the cardiac rhythm becomes dominant [191] as observed in our, and other, adult-based studies.

### 5.6 Conclusion

We conclude that, in resting subjects, there are gender-based differences in the extent to which the respiratory system is able to “drive” the cardiac system, with females displaying much higher levels of coupling and directionality. The increased synchronization seen in older females may result from the maintained strength of coupling between these two systems compared to the case for males, along with the tendency for the RF of females to increase with age. In summary, the data provide definitive information about the central importance of the respiratory oscillator in the synchronization between itself and the cardiac oscillator in the resting state. The duration of the synchronization observed increases as the average RF increases, and is inversely correlated with the variability in the respiratory rate. There are many studies of HRV, its links to autonomic control and its potential use as a diagnostic tool (see for example, [92,192]). We conclude, however, that RF and RFV are also critical indicators of the cardio-respiratory interaction, and that future studies should consider them in more detail.

There are significant gender-specific differences in how the extent of synchronization varies with age. These differences are significantly associated not with changes in the individual oscillators but with alterations in the average RF, and the gender-based differences in the extent of coupling are at least partially involved in the increased synchronization observed in older females.

## 6 Conclusion

Some of the physiological processes associated with ageing have been illuminated by the application of methods drawn from statistical physics and nonlinear dynamics to the analysis of cardiovascular time series.

Two approaches to the analysis of cardiovascular signals have been presented, based on coupled oscillators and statistical mechanics. The observation of cardio-respiratory synchronization demonstrates a well-known property of nonlinear coupled oscillators (Section 5). These results illustrate the fact that the cardiovascular system may be represented by a set of coupled oscillators of relatively few degrees of freedom. At the same time, however, the system is always exposed to noise from unpredictable sources bringing in many additional degrees of freedom. In this sense, the statistical approach, reviewed in Section 3, is useful: although DFA is usually regarded as a way of obtaining a scaling for a system with many degrees of freedom, we showed that there is a close connection between DFA results and the oscillatory components detected using wavelet analysis. Scaling properties, and their connections with coupled oscillator models with relatively few degrees of freedom (but in the presence of noise), clearly deserve further investigation.

Our main conclusions are:

(1) The standard deviation of the heart rate decreases significantly with age for both males and females. The new results presented above are consistent with previous studies [7,38,39,41,120].
(2) The total energy of HRV decreases with age because the contributions to variability attributable to respiratory activity (energy interval II) and to myogenic activity (interval III) decrease with age. Significant decreases with age in both the total energy, and the energy in intervals II and III, were observed in [7]. We found no significant age-related change in the recently identified interval VI. Neither did we observe the significant decreases in intervals III and IV that were reported in [7]; the difference may be associated with the smaller number of subjects in the latter study. It is reported for the first time that females have stronger RSA than males in the younger population.
(3) The complexity of HRV within the range of 10 to 50 s decreases significantly with age. The decrease arises because the ratio of the energy in the slower oscillations of interval IV (neurogenic activities) to the faster oscillations of interval III (myogenic activities) increases significantly with age. This indicates that the neurogenic control of HR becomes more prominent than myogenic control with increasing age. Decreases with age of complexity in HRV were reported in earlier studies such as [44,49]. The difference between the present review and the latter work is the window size used for the calculations. Our window size was determined by the time scales selected using wavelet analysis. Thus we were able to identify for the first time the physiological reasons underlying the decrease of complexity with age.
(4) In this way, the connection was established between complexity analysis and the analysis of oscillatory dynamics. The time scales detected using wavelet analysis help to determine the window size for complexity analysis, and enable us to interpret the results of the complexity analysis.
(5) The responses of young females to the endothelial-related vasodilator ACh are significantly higher than those of young males and aged females, whereas there is no significant gender or ageing-related difference for SNP. It was already known that there is a decrease in endothelial-dependent vasodilation with age and gender difference, from using iontophoresis-stimulated blood flow measurements [47,48,106,107]. Our application of the wavelet transform to such signals has revealed age- and gender-related changes in the oscillatory dynamics for the first time. This indicates that the endothelial function of females is higher than that of males, which may be connected with the well-known fact that young females have lower cardiovascular risk compared with aged females and males.
(6) The duration of cardio-respiratory synchronization epochs increases significantly with age for females. The logarithm of the synchronization duration has a significant correlation with the average respiratory rate and total energy of respiration for both males and females. In addition, respiratory rates affect the synchronization duration exponentially. Earlier studies have reported synchronization under a range of different conditions, e.g. for anæsthetized rats [29], young healthy athletes [27,28], infants [171], healthy adults [32,172–174] and heart transplant patients [32]. The present review is the first to draw attention to the effects of ageing on synchronization and to the strong correlation between the duration of synchronization and the respiratory rate.

From all these results, we conclude that ageing is a significant factor affecting cardiovascular dynamics in healthy individuals, and that gender sometimes produces a significant difference as well. We note that, with the use of a larger subject cohort to improve the statistics, the approaches discussed here could in principle be used to create a basis for quantifying a subject’s “cardiovascular age”. This could be a useful parameter for clinical purposes, for planning and for optimization of quality of life, especially as the measurements concerned are relatively brief, non-invasive and involve no discomfort.

## Figures and Tables

**Fig. A.1:**
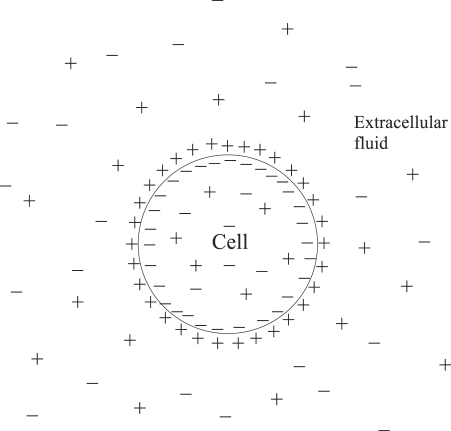
Schematic diagram showing the distribution of charged particles inside and outside the cell membrane.

**Fig. A.2:**
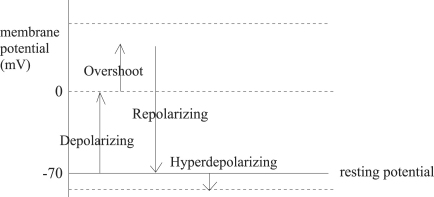
Schematic diagram to illustrate the terminology of membrane potential changes.

**Fig. A.3:**
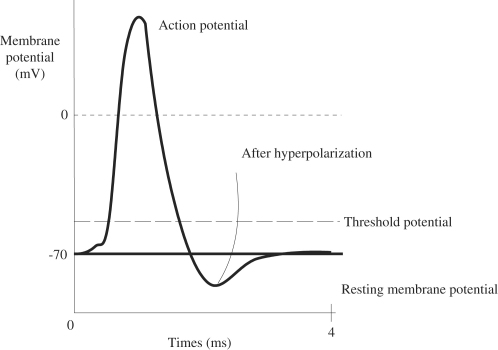
Changes in the membrane potential when an action potential is generated.

**Fig. A.4:**
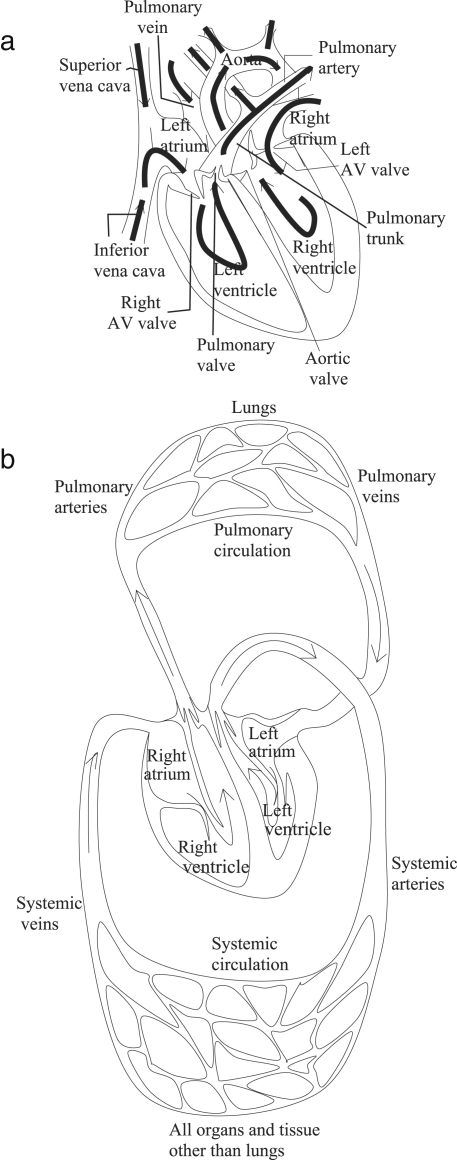
(a) Sketch of the heart, showing its main components. (b) Schematic diagram showing the systemic and pulmonary circulation.

**Fig. A.5:**
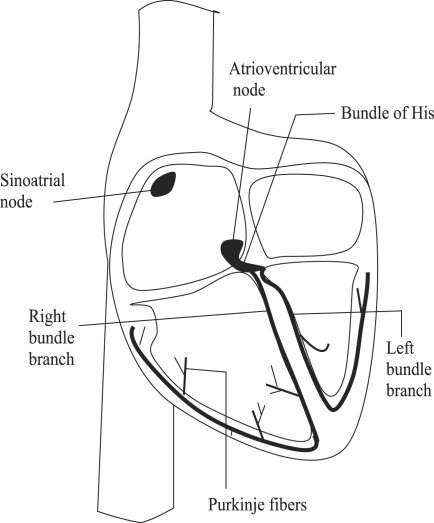
Conducting system of the heart.

**Fig. A.6:**
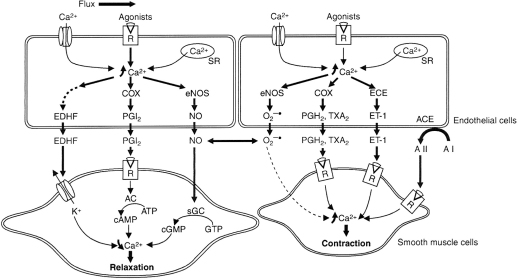
Release of relaxing and contracting factors from endothelial cells and their effects on vascular smooth muscles. AC = adenylyl cyclase; ACE = angiotensin converting enzyme; ATP = adenosine triphosphate; A I = angiotensin I; A II = angiotensin II; cAMP = cyclic adenosine monophosphate; **cGMP** = cyclic guanosine monophosphate; COX = cyclo-oxygenase; ECE = endothelin converting enzyme; EDHE = endothelium-derived hyperpolarizing factor; eNOS = endothelial nitric oxide synthase; ET-1 = endothelin-1; GTP = guanosine triphosphate; NO = nitric oxide; $O_{2}^{-•}$ = superoxide anions; ${\mathrm{PGH}}_{2}$ = prostaglandin $H_{2}$; ${\mathrm{PGI}}_{2}$ = prostacyclin; R = receptor; sGC = soluble guanylyl cyclase; SR = sarcoplasmic reticulum; ${\mathrm{TAX}}_{2}$ = thromboxane $A_{2}$.

This figure is taken from [193].

**Fig. B.1:**
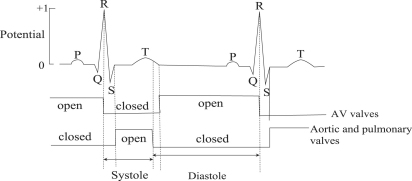
Schematic diagram showing the structure of the ECG cycle and the timing of the associated events occurring in the heart.

**Fig. B.2:**
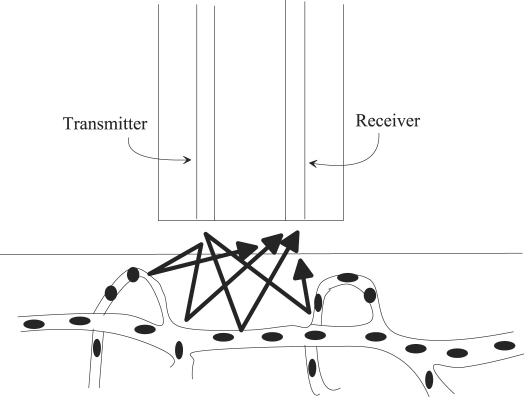
Sketch showing how a laser Doppler flowmetry (LDF) probe detects flow.

**Fig. B.3:**
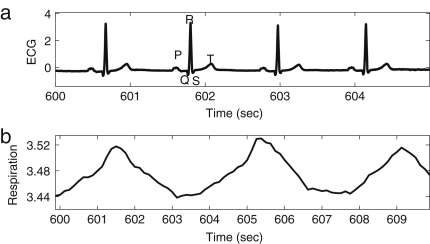
(a) An ECG signal, and (b) a respiratory signal obtained from the measurements. The PQRST events are marked in (a).

**Table C.1:** Values of two groups, considered as an example.

Types	Number	Values										
X	11	33	14	12	11	22	28	10	8	18	19	20
Y	7	17	28	15	35	27	32	29				

**Table C.2:** The ranks assigned to each sample.

Values	8	10	11	12	14	15	17	18	19
Types	X	X	X	X	X	Y	Y	X	X
Ranks	1	2	3	4	5	6	7	8	9

Values	20	22	27	28	28	29	32	33	35

Types	X	X	Y	X	Y	Y	Y	X	Y
Ranks	10	11	12	13.5	13.5	15	16	17	18

The figure is taken from [44].

The figure is taken from [44].

The figure is taken from [128].

**Table 1:** The frequency intervals seen in blood flow oscillations, and their physiological origins.

Interval	Frequency (Hz)	Physiological origin
I	0.6–2.0	Cardiac activity
II	0.145–0.6	Respiration
III	0.052–0.145	Myogenic activity
IV	0.021–0.052	Neurogenic activity
V	0.0095–0.021	Endothelial metabolic activity
VI	0.005–0.0095	Endothelial activity

**Table 2:** Significance of gender differences in interval II of the blood flow wavelet analysis. Cases where females have significantly higher energy than males are indicated with an (f). The first row shows p-values calculated using the Wilcoxon rank sum test, the second row gives the average and standard deviation for males, and the third row gives the average and standard deviation for females.

Gender difference (HRV)				
	Absolute energy		Relative energy	
	Below 40 years	Above 55 years	Below 40 years	Above 55 years
II	p=0.01 (f)	p=0.02 (f)	p=0.05 (f)	p=0.39
Average (male)	0.07±0.06	0.02±0.02	0.29±0.14	0.25±0.20
Average (female)	0.13±0.08	0.06±0.06	0.37±0.15	0.26±0.10

**Table 3:** Significance of the differences in absolute oscillation energy in intervals VI and V between ACh- and SNP-influenced blood flow signals. Cases where the energy in ACh-influenced ones is significantly the higher are indicated with an (A).

Differences in absolute oscillation energy between ACh and SNP				
	Males		Females	
	Below 40 years	Above 55 years	Below 40 years	Above 55 years
Interval VI	p=0.00 (A)	p=0.24	p=0.00 (A)	p=0.00 (A)
Average (ACh) $*10^{3}$	3.13±3.16	3.36±5.08	7.67±6.07	2.65±2.11
Average (SNP) $*10^{3}$	1.99±4.31	0.78±0.70	1.50±3.24	0.52±0.50
Interval V	p=0.04 (A)	p=0.92 intervals	p=0.00 (A)	p=0.09
Average (ACh) $*10^{3}$	1.23±1.14	1.37±1.83	2.45±1.82	0.88±0.53
Average (SNP) $*10^{3}$	0.64±0.43	0.94±0.84	0.88±0.77	0.62±0.56

**Table 4:** Significance of gender differences for the absolute energy in intervals VI and V for ACh (left hand side) and SNP (right hand side). Cases where females exhibited significantly higher energy than males are indicated with an (f). The averages and standard deviations are shown in Table 3.

Gender difference for absolute energy				
	ACh		SNP	
	Below 40 years	Above 55 years	Below 40 years	Above 55 years
Interval VI	p=0.00 (f)	p=0.20	p=0.53	p=0.23
Interval V	p=0.01 (f)	p=0.39	p=0.79	p=0.19

**Table 5:** The differences between the relative energies for ACh- and SNP-influenced blood flow oscillations in frequency intervals VI and V. Cases where the relative energy is significantly higher with ACh are indicated with an (A).

The difference between ACh and SNP for relative energy				
	Males		Females	
	Below 40 years	Above 55 years	Below 40 years	Above 55 years
Interval VI	p=0.00 (A)	p=0.00 (A)	p=0.00 (A)	p=0.00 (A)
Average (ACh)	0.41±0.21	0.27±0.20	0.57±0.18	0.31±0.16
Average (SNP)	0.13±0.10	0.09±0.07	0.17±0.13	0.07±0.05
Interval V	p=0.94	p=0.25	p=0.90	p=0.27
Average (ACh)	0.15±0.05	0.12±0.06	0.17±0.04	0.12±0.07
Average (SNP)	0.16±0.07	0.10±0.06	0.18±0.07	0.10±0.06

**Table 6:** Gender difference between the relative energies in intervals VI and V for ACh-influenced (left hand side) and SNP-influenced signals (right hand side). The case where females exhibit significantly higher energy than males is marked with an (f). The averages and standard deviations are shown in Table 5.

Gender difference for relative energy				
	ACh		SNP	
	Below 40 years	Above 55 years	Below 40 years	Above 55 years
Interval VI	p=0.01 (f)	p=0.51	p=0.19	p=0.70
Interval V	p=0.13	p=0.73	p=0.54	p=0.95

## Appendix A Physiological background

In this appendix we provide a succinct summary of the relevant physiological background needed by non-biological scientists to study the review.

### A.1 Electrical activity in the cell

First, we describe briefly the electrical activity in a single cell, thus providing the basic information for the subsequent sections.

The predominant solutes in the extracellular fluid are sodium and chloride ions. The intracellular fluid also contains high concentrations of potassium ions and ionized non-diffusible molecules, particularly proteins, with negatively charged side chains and phosphate compounds. Electrical phenomena result from the distribution of these charged particles and occur at the cell plasma membrane.

All cells under resting conditions have a potential difference across their plasma membranes. The inside is negatively charged with respect to the outside. This potential difference is known as the membrane resting potential.

By convention, the extracellular fluid is assigned a voltage zero, and the polarity of the membrane potential is stated in terms of the sign of the excess charge inside the cell. The magnitude of the resting potential lies in the range between −5 and −100 mV, depending on the type of cell. The resting potential is steady unless a movement of charged particles occurs between the inside and the outside. The distribution of charged particles inside and outside the cell is shown schematically in Fig. A.1.

Transient changes in the membrane potential from its resting level produce electrical signals. These signals occur in two forms, from graded potentials and action potentials. Graded potentials are important in producing signals that propagate over short distances whereas action potentials propagate over long distances. Graded potentials arise in all cells, but action potentials do not. The latter need some specific function in the membrane. Here we refer only to the action potential because it bears on the following sections.

Before proceeding further, we note that the resting membrane potential also fluctuates in all cells. However, these fluctuations are meaningful only *in vivo*–when a cell is surrounded by other cells and the flow of ions is continuous–whereas the present understanding of the electric properties of membranes comes from *in vitro* studies. We can further hypothesize that the fluctuations in question are most probably related to the fluctuations in endothelial activity, and may perhaps be involved in the endothelial-related oscillations discussed earlier. This possible relationship remains to be explored and elaborated in future research.

The terms depolarize, repolarize, and hyperpolarize are used to describe the direction of changes in the membrane potential relative to the resting potential (Fig. A.2). The membrane is said to be depolarized when its potential is less negative than that of the resting state. Overshoot is a reversal of the membrane potential polarity. When a membrane potential that has been depolarized returns toward the resting value, it is said to be repolarizing. The membrane is hyperpolarized when the inside potential is more negative than the resting level.

Action potentials are rapid and large alterations in the membrane potential (Fig. A.3). They can occur at the rate of 1000 per second. Membranes that are able to produce action potentials are called excitable membranes and the ability to generate action potentials is called excitability. Action potentials occur in response to stimuli. Only when the stimulus is strong enough to cause the membrane potential to reach the threshold potential does it trigger an action potential.

### A.2 The circulatory system

The heart, the vascular system (blood vessels) and the blood are the three principal components that make up the circulatory system. As reported by the British physiologist William Harvey in 1628, the cardiovascular system forms a closed loop, so blood pumped out of the heart through one set of vessels returns to the heart via a different set. The whole system can be divided into two circuits, the pulmonary circulation and the systemic circulation (Fig. A.4b). Each of them starts and ends in the heart. The right and left sides of the heart each have two chambers: the upper chamber is the atrium and the lower one is the ventricle. There is flow from the atrium to the ventricle on each side of the heart but there is no direct flow between the two atria or two ventricles.

The pulmonary circulation causes blood to be pumped from the right ventricle through the lungs and thence to the left atrium. It is then pumped through the systemic circulation from the left ventricle, through all the organs and tissues of the body except the lungs, and thus to the right atrium. In both circuits, the vessels which carry blood away from the heart are called arteries and those which carry blood towards the heart are called veins.

In the systemic circuits, blood leaves the left ventricle via a single large artery, the aorta (Fig. A.4a). The arteries of the systemic circulation branch off the aorta, dividing into smaller vessels. The smallest arteries branch into arterioles, which branch into a huge number of very small vessels, the capillaries. The capillaries then unite to form vessels of larger diameter, the venules and then veins. The flow in arterioles, capillaries and venules is termed microcirculation.

In the pulmonary system, blood leaves the right ventricle via a single large artery, the pulmonary trunk, which divides into two pulmonary arteries, one supplying the right lung and the other the left. In the lungs, the arteries continue to branch and form the capillaries that unite into venules and then veins. The blood leaves the lungs via four pulmonary veins, which empty into the left atrium.

As blood flows through the lung capillaries, it picks up oxygen supplied to the lungs by breathing. So the blood in the pulmonary veins, the left side of the heart, and the systemic arteries has a higher oxygen content. Correspondingly, the blood in the other side of the circulatory system has a lower oxygen content.

Because of the crucial role of the lungs in supplying the oxygen that is then transported by the circulatory system, the lungs can be viewed as a fourth component of the cardiovascular system. The pressure difference that they provide is also important, especially for the venous return, and the lungs can therefore be seen as a second pump, in addition to the heart, within the cardiovascular system.

### A.3 The heart

#### A.3.1 Anatomy

The heart is a muscular organ enclosed in a fibrous sac, the pericardium, and located in the chest. The walls of the heart are composed primarily of cardiac muscle cells and are termed the myocardium. The inner surfaces of the cardiac chambers, as well as the inner walls of the blood vessels, are lined by a thin layer of cells known as endothelium.

Located between the atrium and ventricle in each half of the heart are the atrioventricular (AV) valves, which permit blood to flow from atrium to ventricle, but not from ventricle to atrium (Fig. A.4a).

The opening and closing of the AV valves is a passive process resulting from pressure difference across the valves. When the blood pressure in an atrium is greater than that in the corresponding ventricle, the valve is pushed open and blood flows from atrium to ventricle. In contrast, when a contracting ventricle achieves an internal pressure greater than that in its connected atrium, the AV valve between them is forced to close.

The openings of the right ventricle into the pulmonary trunk, and of the left ventricle into the aorta, also contain valves, the pulmonary and aortic valves, respectively (Fig. A.4a). These valves permit blood to flow into the arteries during ventricular contraction but prevent blood from moving in the opposite direction during ventricular relaxation. They also act in a passive way like the AV valves, and they are either open or closed depending on the pressure differences across them.

#### A.3.2 Heartbeat coordination

The heart is a dual pump in that the left and right sides of the heart pump blood separately but simultaneously. The atria contract first, followed almost immediately by the ventricles. Contraction of cardiac muscle is triggered by depolarization of the plasma membrane. The gap junctions that connect myocardial cells allow the action potential to spread from one cell to another. Thus, the excitation of one cardiac cell results in the excitation of all the cardiac cells. This initial depolarization normally arises in a small group of cells, the sinoatrial (SA) node, located in the right atrium near the entrance of the superior vena cava (Fig. A.5). The action potential spreads from the SA node throughout the atria and then throughout the ventricles. So the SA node works as the pacemaker for the entire heart, and its discharge rate determines the heart rate, the number of times the heart contracts per minute.

The action potential initiated in the SA node spreads throughout the right atrium, and from the right atrium to the left atrium, so rapidly that the two atria contract at the same time.

The spread of the action potential to the ventricles involves the rest of the conducting system, a portion of which is called the atrioventricular (AV) node. The AV node is located at the base of the right atrium (Fig. A.5). The action potential spreading through the right atrium causes depolarization of the AV node. Because the propagation of the action potential through the AV node is relatively slow, atrial contraction is completed before ventricular excitation occurs.

After leaving the AV node, the impulse enters the interventricular septum between ventricles. This pathway has conducting-system fibres termed the bundle of His (Fig. A.5). The AV node and the bundle of His constitute the only electrical link between the atria and ventricles.

Within the interventricular septum the bundle of His divides into right and left bundle branches, which leave the septum to enter the walls of both ventricles (Fig. A.5). These fibres contact with Purkinje fibres, large conducting cells that rapidly distribute the impulse throughout much of the ventricles. Finally the Purkinje fibres make contact with ventricular myocardial cells, by which the impulse spreads through the rest of the ventricles.

#### A.3.3 The mechanical event of the cardiac cycle

The cardiac cycle is divided into two major phases, both named to reflect events that occur in the ventricles. The period of ventricular contraction and blood ejection is called systole, and it alternates with a period of ventricular relaxation and blood filling, diastole. On average, one cardiac cycle lasts approximately 1.0 s, with 0.4 s in ventricular systole and 0.6 s in ventricular diastole.

Both systole and diastole can be subdivided into two periods. During the first part of the systole, the ventricles are contracting but all the valves in the heart are closed and no blood can be ejected. This period is termed isovolumetric ventricular contraction because the ventricular volume is constant. The ventricular walls are developing tension and squeezing the blood that they enclose.

Once the rising blood pressure in the ventricles exceeds that in the aorta and pulmonary trunk, the aortic and pulmonary valves open, and the ventricular ejection period, systole, occurs.

During the first part of diastole, the ventricles begin to relax and the aortic and pulmonary valves close, as do the AV valves too. No blood is entering and leaving the ventricles and the ventricular volume is not changing. This period is therefore called isovolumetric ventricular relaxation. The AV valves then open and ventricular filling occurs as blood flows in from the atria. Atrial contraction occurs at the end of diastole after most of the ventricular filling has taken place.

#### A.3.4 Control of the heart rate

The isolated heart, disconnected from the nervous system, beats approximately at a rate of 100 beats/min. The heart rate in the body may be much lower or higher than this; the SA node is usually under the influence of nerves and hormones. A large number of parasympathetic and sympathetic fibres end on the SA node. Activity of the parasympathetic nerves causes the heart rate to decrease whereas that of sympathetic nerves causes the heart rate to increase. In the resting state, there is considerably more parasympathetic activity to the heart than sympathetic, so the normal resting heart rate of about 60 beats/min is well below the inherent rate of 100 beats/min.

### A.4 The vascular system

The functional and structural characteristics of the blood vessels change with successive branching. But there is one structural component in common in the entire cardiovascular system. It is a smooth single-celled layer of endothelial cells or endothelium, which lines the inner surface of the vessels. Capillaries consist only of endothelium, whereas all other vessels have additional layers of connective tissue and smooth muscle.

#### A.4.1 The endothelium

The endothelium lines the inner walls of blood vessels; the capillaries consist only of endothelium. The endothelial cells are in close contact with each other, and form a layer preventing blood cells from interacting with the vessel wall as blood moves through the lumen. The endothelium plays a critical role in the mechanics of blood flow, the regulation of coagulation, leukocyte adhesion, and vascular smooth muscle cell growth, and it also serves as a barrier to the transvascular diffusion of liquids and solutes.

It was first reported by Furchgott and Zawadzki in 1980 that the intact endothelium produces a factor which causes relaxation of vascular smooth muscle. This was originally named endothelium-derived relaxing factor (EDRF); it is now known to be nitric oxide (NO). Nitric oxide is released continuously by endothelium in the arterioles and contributes to arteriolar vasodilation in the basal state. The production of NO can be stimulated by a variety of endothelial antagonists, including acetylcholine, as well as by shear stress resulting from an increase of blood flow or pressure. In addition to NO, the endothelium releases other vasodilators such as prostacyclin (${\mathrm{PGI}}_{2}$) and vasoconstrictors such as endothelin-1 (ET-1) [193] (see Fig. A.6).

#### A.4.2 Vascular smooth muscle

The smooth muscle cells are arranged in helical or circular layers around the larger blood vessels and in a single layer around arterioles. Vascular muscle cells provide active tension in the vessel wall and regulate the diameter of the vessels.

In many vessels there are smooth muscles that undergo spontaneous depolarization. There cells act as pacemakers and excite neighbouring cells, thus providing background tension, the myogenic basal tone. Their activities are independent of innervation. The property is similar to that of the heart, but the contractile characteristics and the mechanisms that cause contraction of vascular smooth muscle are very different from those for cardiac muscle. Vascular smooth muscle undergoes slow, sustained, tonic contractions, whereas cardiac muscle contractions are rapid and of relatively short duration (a few hundred milliseconds).

Contraction in vascular smooth muscle can be initiated by mechanical, electrical, or chemical stimuli. Passive stretching of vascular smooth muscle can cause contraction that originates from the smooth muscle itself and is therefore termed a myogenic response. Electrical depolarization of the vascular muscle cell membrane also elicits contraction, most likely by opening voltage-dependent calcium channels and causing an increase in the intracellular concentration of calcium. Finally, a number of chemical stimuli such as norepinephrine, angiotensin II, vasopressin, and endothelin-1 can cause contraction. Each of these substances binds to specific receptors on the vascular smooth muscle cell (or to receptors on the endothelium), which then leads to vascular smooth muscle contraction.

### A.5 The nervous system

#### A.5.1 Innervation of the heart

There are two divisions of the autonomic nervous system which affect the heart’s activities: the parasympathetic nervous system and the sympathetic nervous system.

Parasympathetic innervation is achieved by the two vagus systems. The right vagus nerve affects the SA node predominantly. This nerve has an ability to slow SA nodal firing and even stop it for several seconds. The left vagus nerve mainly inhibits AV conducting tissues. The sympathetic nerve supply is nearly uniformly distributed in the heart. Increased sympathetic activity produces an increase in the heart rate and velocity and force of contraction.

As mentioned above, both divisions of the autonomic nervous system have a tonic influence on the cardiac pacemaker, the SA node. The sympathetic nerve system enhances the autorhythmicity, whereas the parasympathetic nervous system inhibits it. Parasympathetic nerves predominate in healthy, resting individuals. After a parasympathetic blockage, the heart rate increases substantially, and after sympathetic blockade, it decreases slightly. After a blockade of both divisions, the heart rate is about 100 beats/min for young adults.

#### A.5.2 Innervation of the vessels

Most of the arteries and veins are innervated by the sympathetic nervous system. The fibres have a tonic contractile effect on the blood vessels.

### A.6 Vasodilatory substances

In this section, details of the substances ACh and SNP are described.

#### A.6.1 Acetylcholine

Acetylcholine (ACh) is a neurotransmitter that is released from axonal terminals, passes across the synaptic connection between two neurons, or between a neuron and a muscle fibre, and contributes to the potential activation of the second neuron or the muscle fibre. Its chemical formula is CH_3_COOCH_2_CH_2_N^+^(CH_3_)_3_. Acetylcholine was first identified in 1914 by Henry Hallett Dale, then confirmed as a neurotransmitter by Otto Loewi in 1921.

It is present in both the autonomic and somatic nervous systems. In the autonomic system it is one among many transmitting substances; it is the only transmitter in striated muscles. When it binds to the acetylcholine receptors of striated muscle fibres, it stimulates those fibres to contract. ACh is also released and used in the brain, where it tends to cause excitatory actions.

In the autonomic system ACh causes the opposite effect to that in striated muscle or brain. An increase in ACh released from parasympathetic fibres, i.e. vagus nerves, causes a decrease in heart rate. Similarly, an increase in ACh in the sympathetic fibres that innervate vessels results in vasodilation.

As noted above in Appendix A.4.1, this vasodilation occurs through the action of the endothelium. The crucial observation was by Furchgott and Zawadzki who showed in experiments on rabbits that the removal of endothelium prevented ACh-induced vasodilation [98].

#### A.6.2 Sodium nitroprusside

Sodium nitroprusside (SNP) breaks down in the blood and releases nitric oxide (NO). The nitric oxide enters the muscle cells in the walls of the blood vessels and causes them to relax. When the muscles relax, the blood vessels become wider and the blood pressure decreases. The chemical formula of SNP is Na_2_[Fe(CN)_5_NO]_2_.

SNP is used for the emergency treatment of high blood pressure (hypertensive crisis). It is also used to produce controlled hypotension (low blood pressure) in anæsthetized patients during surgery. It had been used in the emergency treatment of severe heart failure to reduce heart workload. However, it has side effects and is no longer used for clinical treatment of hypertension.

## Appendix B Physiological measurements

### B.1 Measurement time

Many different rhythmical processes occur in living systems [194], and they manifest on widely differing time scales. Examples include predator–prey cycles (with a period typically of years), seasonal growth/decay (a year), sleep-wakefulness and other circadian rhythms (24 h), tidal rhythms in marine life (12 h), respiration (10 s), heartbeat (1 s), and EEG brainwaves (milliseconds to seconds). For studies of any rhythm, the measurement/observation time should always be made considerably longer than the time scale of the phenomenon under study. In the case of the work considered in the present review, interest centres on phenomena that occur on the scale of the blood distribution time. Under steady conditions in resting subjects, the volume of blood pumped by the heart in one minute is on average equivalent to the whole amount of blood in the organism [195]. Thus, the dynamics of the blood distribution can be analysed on a time scale of minutes. We are especially interested in oscillations whose frequencies lie in the range 0.005 to 2.0 Hz, i.e. of period 0.5–200 s. Their physiological origins have been described in Section 4.3.1.

If a signal is perfectly periodic, one period is enough to specify it. However, signals from the human cardiovascular system are not periodic, but quasiperiodic, and their periods fluctuate constantly. Consequently, the measurement should be long enough to contain at least several periods. On the other hand, the longer a signal is, the more pronounced the effects of non-stationarity become. For example, changes of physiological conditions, or physical movement, can occur during measurements. In our own measurements we chose 30 min as a compromise that would still allow us to study the slowest oscillations of interest (interval VI), with a period of ∼200 s.

### B.2 Measurement techniques

#### B.2.1 ECG

The electrocardiogram (ECG) is a tool for evaluating the electrical events in the heart. ECG measurements have been used for diagnostic purposes for more than a century. The action potentials of cardiac muscle cells can be viewed as batteries that cause charge to move throughout the body fluids. These moving charges are caused by all the action potentials occurring simultaneously in many individual myocardial cells, and their result can be detected by recording electrodes on the surface of the skin. The ECG does not provide a direct record of the changes in membrane potential across individual muscle cells. Rather, it is a measure of the currents generated in the extracellular fluid by the changes occurring simultaneously in many cardiac cells.

A conventional three-lead measurement of the ECG is performed by using three leads, placed on the right and left shoulder bones for the first and second leads, and on the left leg or lower left rib bone for the third lead. The resultant ECG signal, recorded in this way, is shown schematically in Fig. B.1.

The P wave is the first deflection and represents the electrical impulse through the atrial musculature (depolarization). The second deflection is the QRS complex and represents the spread of the electrical impulse through the ventricular musculature, which triggers the ventricular contraction. The P–R interval is measured from the beginning of the P wave to the beginning of the QRS complex. It reflects the time taken by impulse to travel the entire distance from the SA node to the ventricular muscle. The final deflection is the T wave and represents the period of recovery for the ventricles (i.e. repolarization).

#### B.2.2 Respiration

Respiration results in a constant exchange of oxygen and carbon dioxide between the organism and its external environment. In humans, the respiratory system includes the oral and nasal cavities, the lungs, the series of tubes leading to the lungs, and the chest structures which move the air into and out of the lungs. In respiration, there are two movements: inspiration and expiration. Inspiration is the movement of air from the external environment into the lungs, enlarging their volume. Expiration is movement in the opposite direction. One cycle of respiration consists of an inspiration and an expiration. In our own recordings, respiration was measured by a belt around the thorax to detect the change of the volume of the lungs (Respiratory effort transducer TP-TSD201, BIOPAC Systems, Inc., Goleta, CA, USA).

#### B.2.3 Blood flow

(a) Laser Doppler flowmetry

After the first laser was demonstrated by Maiman [196], Cummins et al. proposed a way of measuring the velocity of particles in solution by using the Doppler frequency shift of back-scattered light [197]. After some years, Riva et al. applied this technique to the measurement of the velocity of red blood cells in a glass-tube flow model [198] and Stern used the laser Doppler technique for blood perfusion measurement in the undisturbed microcirculation [199].

The laser Doppler technique is illustrated schematically in Fig. B.2. It measures blood flow in the very small blood vessels of the microvasculature, such as the low speed flows associated with nutritional blood flow in capillaries close to the skin surface and flow in the underlying arterioles and venules involved in the regulation of skin temperature. The tissue thickness sampled is typically 1 mm, the capillary diameters 10 μm and the velocity spectrum measurement typically 0.01 to 10 mm/s. The technique depends on the Doppler principle by which low power light from a monochromatic (single-wavelength) stable laser is scattered by moving red blood cells and, as a consequence, has its frequency broadened. The frequency-broadened light, together with laser light scattered from static tissue, is photodetected and the resulting photocurrent processed to provide a blood flow measurement. Thus there are two optical fibres in laser Doppler probes. One is to deliver light to the tissues and the other is to collect the scattered light. Bonner and Nossal showed that after filtering the scattered light, they could obtain an output that was proportional to the velocity and the number of red blood cells in the measured volume, but which was unrelated to the direction of movement of the red blood cells [200].

The corresponding instruments for blood flow measurement were developed by Watkins and Holloway [201], Nilsson et al. [202] and Fischer et al. [203]. They demonstrated a good correlation between the output of the devices and the blood flow.

In our study, based on a Moor Instruments (Axminster) DT4 instrument, a near-infra-red laser of wavelength 785 nm is used to measure the velocity and concentration of red blood cells within the volume covered by the laser light. The size of the probed volume depends on several factors: the optical density of the tissue, the separation of the probe fibres, and the power and wavelength of the laser. From imaging at this wavelength with the DRT4, it is known that the full dermal thickness (about 1 mm) is probed, so from simple geometry a hemisphere of radius 1 mm gives a volume of approximately 2 mm^3^.

Because there is a residual value called the *biological zero*, even in the case of complete occlusion [5], we cannot use absolute units (e.g. ml/s/mm^3^) but are obliged to work in arbitrary units (AU) for the flow. The residual value comes from the Brownian motion of the red blood cells. To obtain an absolute measure, the biological zero is determined by doing probe calibration in water containing a colloidal suspension of polystyrene microspheres of known characteristics.

(b) Iontophoresis

Iontophoresis is widely used for transcutaneous delivery of ionized drugs for the assessment of skin microvascular function. A small electrical current is used to transfer locally vasoactive substances such as endothelial-dependent ACh and endothelial-independent SNP across the dermal barrier as unipolar currents of relatively large molecules. It has been shown [204] that the potential difference applied does not in itself cause a significant increase in blood flow. As discussed in Appendix A.6.2, SNP breaks down to yield nitric oxide (NO), which acts directly on vascular smooth muscle cells, while ACh acts on the intact vascular endothelium causing it to release NO. Endothelial activity can therefore be assessed by looking at the difference between ACh- and SNP-stimulated oscillations. In this way oscillations around 0.01 Hz and 0.07 Hz were shown to be endothelial dependent [99–101,103]. ACh and SNP are relatively high conductivity solutions and have opposite polarity. Therefore we need to apply anodal iontophoresis to ACh and cathodal iontophoresis to SNP.

However, there are some potential methodological problems related to iontophoresis. It is known that blood flow can sometimes be increased in response to the current itself, in the absence of vasoactive drugs but just with pharmacologically neutral electrolytes such as H_2_O[205,206] or NaCl solution [207]. This phenomenon is known as the *galvanic effect*, or *current-induced vasodilation*. The mechanism is unclear but could involve, for example, local heating due to the voltage required to convey the ions through the dermal barrier. It was shown in [113] that the magnitude of voltage between the adjacent chambers needed to sustain the chosen iontophoresis current is not an important factor in causing changes in blood flow using H_2_O and NaCl. In another earlier study [208] H_2_O and NaCl were used with exactly the same protocol as is described in this review, with both anodal and cathodal iontophoresis. The results indicated that the differing responses of the 0.01 Hz spectral component to ACh and SNP may be interpreted with confidence as a specific effect of the substances, and not of the iontophoresis current itself.

Currents were delivered from a battery-powered constant current iontophoresis controller (Moor Instruments MIC1-e). The iontophoresis probe-holders were of Perspex with internal platinum wire electrodes. Their internal diameter was 8 mm, giving an area of 1 cm^2^ in contact with the skin. The dosages of the drugs delivered are proportional to the total charge (Q) in millicoulombs (mC) which migrates through the skin surface, determined by the product of constant current measured in milliamperes (mA) and the duration (t) of the current flow in seconds. We used a protocol that passes a charge of 2 mC (100 μA for 20 s) followed by a 240 s interval seven times in one measurement, thus filling the 30 min of recording.

#### B.2.4 The measurement set-up

The measurement of two blood flow channels with ACh and SNP was obtained by laser Doppler flowmetry (LDF) with a commercially available instrument (DRT4, Moor Instruments, Axminster, Devon, UK). A battery-powered constant current source for iontophoresis was connected to the DRT4 and the probe-holders for ACh and SNP. The two iontophoresis chambers combined with laser Doppler MP1 probes were positioned on the anterior side of the left forearm on adjacent sites under which the vessel densities were similar. A 1% solution of electrolyte was placed in each chamber.

The measurement of basal blood flow was obtained with two additional probes (MoorLAB server and MoorLAB satellite, Moor Instruments, Axminster, Devon, UK). They were fixed on the right wrist and on the inner right ankle respectively.

The cut-off frequency of the low pass filter of 22.5 kHz and a time constant of 0.1 s were selected, thus allowing the dynamics of the slow oscillatory processes to be captured.

The ECG was set up as described in Appendix B.2.1. The ECG and respiration signals were amplified by a signal conditioning unit (Cardiosignals, Jožef Stefan Institute, Ljubljana, Slovenia). All signals were sampled at 400 Hz using a 16-bit A/D converter (National Instruments) and stored in a personal computer.

### B.3 Subjects

In the course of data recording, 118 healthy individuals were studied, including 71 males (42.6±15.1 years, range 16–74 years) and 47 females (45.8±16.6 years, range 18–82 years). They were not taking any medication, nor did they have any history of cardiovascular disease prior to the recordings. They were asked to refrain from eating or drinking coffee for one hour before the measurements.

Subjects lay on a bed in a supine position and were asked to relax while their peripheral blood flow, ECG and respiration were recorded throughout 30 min. The measurements were done at room temperature, 22±1 ^∘^C, during daytime.

### B.4 Recorded signals

#### B.4.1 The ECG and respiration

For the first few decades of ECG measurements, attention was focused mainly on the detailed *shape* of the approximately periodic pulses seen in the signal. As discussed in Appendix B.2.1, a typical ECG signal consists of the P wave, the QRS complex, and the T wave as illustrated in Fig. B.3a. A typical respiratory signal is shown in Fig. B.3b. The maximum point in each period represents inspiration and the minimum expiration.

#### B.4.2 Blood flow with iontophoresis

Blood flow signals with iontophoresis are shown in Fig. B.4. Parts (a) and (b) show the full record of 30 min for ACh and SNP respectively. Part (c) shows the pulses of iontophoresis current. In parts (d) and (e), just 10 s of the records are shown in order to resolve the faster oscillation. Note that the flow and its oscillations increase by a factor of more than 10× after applying the substances.

## Appendix C Statistics

Physiological signals taken from different subjects usually have variance in their characteristics. It is of course inevitable, because there are many possible unknown factors which could be responsible for individual differences. In order to judge the trend of signals, a careful application of statistics is needed. In this Appendix, we explain in detail the statistical methods that have been used in the main part of the review.

Standard tests have been used to determine the statistical significance of the observations. When the underlying distribution of the data was known, or could be assessed, a parametric test was conducted, e.g. a t-test [209]. It can be used to determine whether two Gaussian populations have different distributions in their statistics. On the other hand, when the underlying distribution of data was unknown, a non-parametric test was conducted: we used the Wilcoxon rank sum test and Spearman’s rank correlation coefficient, because we had no prior knowledge of the distribution of samples. In Appendix C.1, the details of the significance tests are explained. Correlation analysis is used to draw inferences about the strength of the relationship between two or more variables. In Appendix C.2, the details of the correlation analysis will be explained.

### C.1 Significance tests

A conjecture concerning the unknown distribution of a random variable is called a statistical hypothesis. The aim of a significance test is to establish whether the hypothesis is true or not. If the probability that the hypothesis holds is below the threshold chosen for statistical significance, the hypothesis is rejected. The statistical significance is usually set to 0.05 and the same value was used in this review. Again, the statistical significance tests were conducted using MatLab (MatWorks).

#### C.1.1 The t-test

In this subsection, a typical parametric test, the t-test, is explained briefly. The t-statistics method was introduced by William Sealy Gosset for cheaply monitoring the quality of stout. Gosset published his t-test in Biometrika in 1908, but was forced to use a pen name, “Student”, by his employer who regarded the fact that they were using statistics as a trade secret.

Given two data sets $X_{1}$ and $X_{2}$, each characterized by its mean $\overset{¯}{X_{1}}$ and $\overset{¯}{X_{2}}$, standard deviation $s_{1}$ and $s_{2}$ and number of data points $n_{1}$ and $n_{2}$, we can use a t-test to determine whether the means are distinct under the assumption that the underlying distributions can be assumed to be normal. All such tests are usually called Student’s t-tests. Strictly speaking that name should only be used if the variances of the two populations are also assumed to be equal. The test used when this assumption is dropped is sometimes called Welch’s t-test. There are different versions of the t-test depending on whether the two samples are independent of each other (e.g., individuals randomly assigned into two groups), or paired so that each member of one sample has a unique relationship with a particular member of the other sample (e.g. the same subjects measured before and after an intervention, or IQ test scores of a husband and a wife).

In the case where two samples are independent, the t-value is calculated as

(C.1) $$t=\frac{\overset{¯}{X_{1}}-\overset{¯}{X_{2}}}{s_{X_{1}-X_{2}}}\text{,}\;\mathrm{where}\;s_{X_{1}-X_{2}}=\sqrt{\frac{\left(n_{1}-1\right){s_{1}}^{2}+\left(n_{2}-1\right){s_{2}}^{2}}{n_{1}+n_{2}-2}\left(\frac{1}{n_{1}}+\frac{1}{n_{2}}\right)}\text{.}$$

Once a t-value is determined, a p-value can be found using a table of values from the t-distribution with ($n_{1}+n_{2}-2$) degrees of freedom. The t-distribution $f_{T}$ is a symmetric bell-shaped distribution with heavier tails than the normal distribution. The t-distribution is defined as

(C.2) $$f_{T}\left(t\right)=\frac{\Gamma \left(\left(k+1\right)/2\right)}{\sqrt{k\pi }\Gamma \left(k/2\right)}{\left(1+t^{2}/k\right)}^{-\left(k+1\right)/2}\text{,}$$

where k is the degree of freedom. The p-value is calculated as

(C.3) $$p=2\int_{t}^{\infty }f_{T}\left(t\right)dt\text{.}$$

If the p-value is below the threshold chosen for statistical significance (usually 0.05), then the null hypothesis $H_{0}$ that the distributions of the two groups are identical is rejected in favour of an alternative hypothesis, which typically states that the groups do differ.

The t-test is also used to examine whether the slope of a regression line differs significantly from 0.

#### C.1.2 The rank sum test

Rank sum tests form a large category of non-parametric tests [144]. The general idea is that, instead of using original observed data, we list the data in ascending order and assign a rank to each item, the position where the item appears in the sorted list. Using the ranks instead of the original observed data makes the test much less sensitive to outliers and noise than parametric tests.

Depending on the number of classes in the data sets, there are different kinds of rank sum tests. The Wilcoxon rank sum test [210] is a non-parametric alternative to the t-test. Here we focus on the Wilcoxon rank sum test and demonstrate how to conduct it by taking an example. Let us take two groups X and Y. X contains 11 samples and Y contains 7 samples as shown in Table C.1. We want to test whether the null hypothesis $H_{0}$ that the distribution of samples X is identical to that of samples Y is true or false.

We combine all the samples of X and Y and sort them into ascending order. Ranks are then assigned to the samples on the basis of their order. If k samples have the same rank of i, then all k samples have an average rank i+(k−1)/2. The results for the example are shown in Table C.2

Suppose that $n_{1}$ and $n_{2}$ are the numbers in the smaller sample and the larger sample, respectively. In this example, $n_{1}=7$ and $n_{2}=11$. Then we calculate the sum for group Y and have the statistic W=6+7+12+13.5+15+16+18=87.5. If the null hypothesis $H_{0}$ holds, the statistic W should be around the expectation value $\left(n_{1}+n_{2}+1\right)\times n_{1}/2=66.5$. If W is too small or too large, the null hypothesis $H_{0}$ is likely to be false. Using MatLab, the p-value is calculated as p=0.0589 for this example. If we set the statistical significance to 0.05, the null hypothesis $H_{0}$ cannot be rejected because 0.05<0.0589, whereas if we set the statistical significance to 0.1, the null hypothesis $H_{0}$ is rejected.

We used the Wilcoxon rank sum test to assess the differences between different genders, different age groups and different drugs.

#### C.1.3 The Wald–Wolfowitz runs test

The Wald–Wolfowitz test is a non-parametric statistical test for checking the hypothesis that the elements of a two-valued data sequence are mutually independent. A “run” is the maximal non-empty segment of the sequence consisting of adjacent elements that are the same. For example, the sequence +−−++−+++−− consists of six runs, three of which consist of +s and the others of −s. Let us suppose that the number of runs is R, the total number of observations is N, $N_{+}$ is that of occurrences of + and $N_{-}$ is that of occurrences of ($N=N_{+}+N_{-}$). In the above example, R is 6, N is 11, $N_{+}$ is 6 and $N_{-}$ is 5. Then the mean μ and standard deviation σ of the probability at which R is obtained given $N_{+}$ and $N_{-}$ are calculated as

(C.4) $$\mu \left(R\right)=1+\frac{2N_{+}N_{-}}{N}$$

(C.5) $$\sigma \left(R\right)=\frac{2N_{+}N_{-}\left(2N_{+}N_{-}-N\right)}{N^{2}\left(N-1\right)}\text{.}$$

 When N is relatively large (N>20), the distribution of R is approximately normal. If there are far more or less runs than expected, the hypothesis of statistical independence of the elements may be rejected. Runs tests can be used to test:

(1) The randomness of a distribution, by taking the data in the given order and marking with + the data greater than the median, and with the data less than the median. (Numbers equal to the median are omitted.)
(2) Whether a function fits well to a data set, by marking the data exceeding the function value with + and the other data with −. For this we use a runs test which takes the signs into account.

We conducted the runs test by using MatLab, which returns values of h and p. This is a test of the null hypothesis that the +s and −s come in random order, against the alternative that they do not. The test returns the logical value h=1 if it rejects the null hypothesis at the 5% significance level, and h=0 if it cannot. The p-value is computed from either the test statistics or the exact distribution of the number of runs and if p is below the significant level (usually 0.05), the null hypothesis is rejected.

### C.2 Correlation analysis

#### C.2.1 Linear correlation

Suppose that we have n observations of two variables, $X=x_{1},\ldots ,x_{n}$ and $Y=y_{1},\ldots ,y_{n}$. The variation of variable Y can be separated into two parts: the variation associated with variable X and the variation not associated with variable X. The fraction that is explained by a linear relationship between X and Y is called the coefficient of determination and its square root is the correlation coefficient. The correlation coefficient r can be expressed [211] as

(C.6) $$r=\frac{\overset{n}{\underset{i=1}{\sum }}x_{i}y_{i}-\frac{1}{n}\left(\overset{n}{\underset{i=1}{\sum }}x_{i}\right)\left(\overset{n}{\underset{i=1}{\sum }}y_{i}\right)}{\sqrt{\overset{n}{\underset{i=1}{\sum }}x_{i}^{2}-\frac{1}{n}{\left(\overset{n}{\underset{i=1}{\sum }}x_{i}\right)}^{2}}\sqrt{\overset{n}{\underset{i=1}{\sum }}y_{i}^{2}-\frac{1}{n}{\left(\overset{n}{\underset{i=1}{\sum }}y_{i}\right)}^{2}}}\text{.}$$

The estimated correlation coefficient r is a random variable that has a distribution function. The distribution of r is a function of the sample size n and the real correlation coefficient ρ. A correlation coefficient of zero means that there is no linear relationship between the two variables. To test whether two variables are linearly related, we set the null hypothesis:

(C.7) $$H_{0}:\rho =0\text{.}$$

It can be shown that for n>2, this hypothesis can be tested using a t-test that is given by

(C.8) $$t_{r}=r\sqrt{\frac{n-2}{1-r^{2}}}\text{.}$$

The value $t_{r}$ is a random value with a t-distribution that has n−2 degrees of freedom. The p-value is defined as

(C.9) $$p=2\int_{t_{r}}^{\infty }f_{T}\left(t\right)dt\text{.}$$

If the p-value is lower than statistical significance, then the null hypothesis is rejected and the correlation coefficient is considered statistically significant.

It is important to check the relationship between variables graphically before performing the correlation analysis in order to check whether there might be an outlier in the data. In the t-test, an outlier can affect the statistics significantly. In some cases, variables have a nonlinear relationship and this can be identified graphically as well. It was reported by Anscombe [212] that several different patterns, one of which had a linear relationship and some of which did not, could return the same correlation coefficient. Nonlinearity may result in low correlation and may sometimes be improved by using a log plot.

#### C.2.2 Spearman’s rank correlation coefficient

Spearman’s rank correlation coefficient or Spearman’s rho, named after Charles Spearman and often denoted by the Greek letter ρ, or sometimes as $r_{s}$, is a non-parametric measure of correlation — that is, it assesses how well an arbitrary monotonic function could describe the relationship between two variables, without making any other assumptions about the particular nature of the relationship between them. It is unaffected by outliers.

Suppose that $X_{i}$ and $Y_{i}$ are two variables between which we want to find the correlation and $x_{i}$ and $y_{i}$ are the rankings of $X_{i}$ and $Y_{i}$ respectively.

(C.10) $$\rho =1-\frac{6\sum {d_{i}}^{2}}{n\left(n^{2}-1\right)}\text{,}$$

where $d_{i}$ is the difference between the two variables, $x_{i}-y_{i}$.

In order to assess the significance level of ρ, we shuffled one variable and obtained ρ from the permuted data. We repeated this process 10^5^ times and calculated the probability of the ρ of the shuffling data being larger than that of the real data. We represented this probability as p and used this value as an indicator of the significance level.

In plotting the relationships of two variables in the main part of the review, we present ρ and p in each figure. If ρ is positive, there is positive correlation, and if ρ is negative, there is negative correlation. However, we do not plot linear regression because we do not assume a linear relationship (unlike for the cases of the Pearson correlation coefficient).

Throughout this review, we used Spearman’s rank test to calculate correlations with age, and their significance, on the basis of the utilization of MatLab (MatWorks). We used $\rho_{m}$ and $p_{m}$ to represent correlation with age and probability for males, respectively, $\rho_{f}$ and $p_{f}$ for females. The blue lines and dots represent males, and the red lines and dots females in the various graphs.
